# Antibiotic resistance modifying ability of phytoextracts in anthrax biological agent *Bacillus anthracis* and emerging superbugs: a review of synergistic mechanisms

**DOI:** 10.1186/s12941-021-00485-0

**Published:** 2021-12-02

**Authors:** Mackingsley Kushan Dassanayake, Teng-Jin Khoo, Jia An

**Affiliations:** 1 School of Pharmacy, Faculty of Science and Engineering, University of Nottingham Malaysia, Jalan Broga, 43500 Semenyih, Malaysia; 2 Singapore Centre for 3D Printing, School of Mechanical and Aerospace Engineering, Nanyang Technological University, Singapore, Singapore

**Keywords:** Phytochemicals, Antibiotic-potentiating, Growth inhibitory indices, Fractional inhibitory concentration index, Superbug bacteria, Anthrax, *Bacillus anthracis*, Antibiotic resistance

## Abstract

**Background and objectives:**

The chemotherapeutic management of infections has become challenging due to the global emergence of antibiotic resistant pathogenic bacteria. The recent expansion of studies on plant-derived natural products has lead to the discovery of a plethora of phytochemicals with the potential to combat bacterial drug resistance via various mechanisms of action. This review paper summarizes the primary antibiotic resistance mechanisms of bacteria and also discusses the antibiotic-potentiating ability of phytoextracts and various classes of isolated phytochemicals in reversing antibiotic resistance in anthrax agent *Bacillus anthracis* and emerging superbug bacteria.

**Methods:**

Growth inhibitory indices and fractional inhibitory concentration index were applied to evaluate the in vitro synergistic activity of phytoextract-antibiotic combinations in general.

**Findings:**

A number of studies have indicated that plant-derived natural compounds are capable of significantly reducing the minimum inhibitory concentration of standard antibiotics by altering drug-resistance mechanisms of *B. anthracis* and other superbug infection causing bacteria. Phytochemical compounds allicin, oleanolic acid, epigallocatechin gallate and curcumin and *Jatropha curcas* extracts were exceptional synergistic potentiators of various standard antibiotics.

**Conclusion:**

Considering these facts, phytochemicals represents a valuable and novel source of bioactive compounds with potent antibiotic synergism to modulate bacterial drug-resistance.

## Background

The emergence of multidrug-resistant bacteria has become a threat to global public health and invoked problems, resulting in inadequate treatment for infectious diseases. The chemotherapeutic management of bacterial infections has become more challenging in recent years due to the development of antimicrobial resistance in pathogenic bacteria and their certain populations evolving into formidable super drug-resistant strains known as superbugs that are capable of causing serious illnesses [1, 2]. The bacterium *Bacillus anthracis* was one of the organisms of interest in the recent past due to its ability to cause the life-threatening illness anthrax. It can be difficult to treat anthrax if progressed to advanced stages, due to the virulence nature of the pathogen and therefore, research has been undertaken to discover novel therapies to treat the disease more safely and effectively [3]. Antibiotics have the ability to restrict the growth and replication of bacteria by inhibiting bacterial cellular components associated with the synthesis of cell walls, proteins and nucleic acids, along with the suspension of folate metabolism and depolarization of the cell membrane [4, 5]. The successful management of bacterial infections has been achieved in the past due to the development of antibiotic agents. It seems, however, that the golden era of these synthetically produced antibiotics has come to a near end due to their irrational use. Although antibiotics have broad-spectrum activities, bacteria have evolved to combat the action of these agents through various resistance mechanisms, such as the production of antibiotic molecule inactivating enzymes, the modification and mutation of antibiotic binding sites, suspension of bacterial cell membrane porin activity associated and the expression of efflux pumps [6, 7]. Another ingenious strategy of overcoming antimicrobials is the deployment of autoinducer molecules by certain bacteria to mediate quorum sensing. Quorum sensing (QS) is a bacterial cell-to-cell biochemical communication process that involves the activation of specific signals to coordinate pathogenic behaviors and assist bacteria to acclimatize against unfavorable conditions imposed by the proximal environment that they exist. Signal molecules responsible for mediating bacterial quorum sensing include autoinducing peptides, autoinducer-2 and acyl-homoserine lactone. Quorum sensing in bacteria can facilitate biofilm formation which agitates the penetration of antibiotic molecules and therefore, it is a major contributor towards antibiotic resistance [8]. Despite the complex nature of issues related to antibiotic resistance, no individual nation has independently succeeded in addressing this major public health problem up to date.

The rise of antimicrobial resistance has mediated interest in research focusing on the significance of medicinal plants and their phytochemical compositions. Long before the invention of modern antibiotics, folklore medicine was able to use the therapeutic efficacy of these medicinal herbs and integrate their potentials in the treatment of infectious diseases. So far, the World Health Organization (WHO) reports that about 80% of the populations residing in Asia and Africa rely on traditional medicine for their primary health care needs [9]. These traditional therapeutic methods have been considered as possibly the safest alternative sources of antimicrobial agents available, by which the involvement of medicinal herbs in treating infectious diseases has paved the way for the development of modern medicine [10]. Plants are reservoirs of chemical agents with therapeutic properties beneficial to mankind [11]. Bioactive compounds naturally extracted from whole plants or from different parts of plants, like leaves, bark, stem, roots, fruits, fruit rind, seeds and flowers, can serve as novel sources for the management of infectious diseases caused by pathogenic microorganisms as an alternative to synthetic drugs [12, 13].

Certain phytochemical compounds are capable to interact synergistically with antibiotics already available, which can be potentially an effective way to combat the phenomenon of resistance. There is evidence that combinations of natural compounds from plants can facilitate or improve the interaction of antibiotics with their target in the pathogen and thus reduce the emergence of resistance through mechanisms of resistance modification [1]. This combined therapeutic strategy can also reduce drug/dose-related side effects to the consumer since lower concentrations of both agents can be used. Therefore, the objectives of this review are to provide an update of the literature review on the synergism between antibiotics and plant extracts, presentation of experimental data on antibiotic-potentiating mechanisms of plant-derived compounds and scientific evidence that support the successive pre-clinical application of synergistic effects of combined plant compounds to serve as a starting point for the discovery of novel antibacterial agents that are capable of neutralizing infections and reversing antibiotic resistance in anthrax causative organism *B*. *anthracis* and the Centers for Disease Control and Prevention (CDC) classified emerging bacterial superbugs.

## Antibacterial drug discovery and development

Long before the twentieth century, the management and treatment of infectious diseases were based mainly on folk medicine. There is evidence that complex mixtures with antibacterial properties have been applied among ancient populations for over two thousand years [14–16]. Post-mortem analysis has revealed the presence of traces of tetracycline like compound that has been incorporated into the dentals of early Sudanese populations that lived around A.D 350–500. The presence of this compound in their corpses has lead to the impression that these populations may have used it as a medicine or included in their diet [17, 18]. A similar finding was reported in ancient populations living in the Dakhleh Oasis, in Egypt, around the time of the late Roman Empire [19]. There is a popular anecdote that showed how to use the red soil in the Hashemite Kingdom of Jordan as a source of antimicrobials to treat skin infections. Interestingly, the bacterium known as Actinomycetes, which is generally found in such soils, produces modern antibiotics, such as actinomycin, streptomycin, erythromycin, nystatin, amphotericin and vancomycin [20, 21]. Traditional Chinese medicine consists of a large summary of medicinal herbs used for millennia in the treatment of many infections caused by bacteria [22, 23]. Their application of active compounds from ancient medicinal herbs has enriched the arsenal of many antibacterial agents used in modern medicine [24]. The modern era of antibacterial agents began with the discovery of penicillin extracted from a mould specimen known as *Penicillium notatum* by Sir Alexander Fleming in 1928. Penicillin caught the attention of many ancient scientists, as the compound was able to stop the growth of a wide range of bacteria [25–27]. During the time of its discovery, penicillin became the most popular therapeutic agent due to the wide application and the magnitude of its therapeutic outcomes. The technologies used and developed to produce penicillin became the basis for the production of all subsequent antibiotics currently in use [28, 29]. Most antibiotics currently in existence, such as cephalosporins, penicillins, macrolides, tetracyclines, vancomycin, teicoplanin, daptomycin and rifamycin have been synthetically derived from natural products [30, 31]. According to the World Health Organization, more than 11% of modern drugs are derivatives from plants [32]. Advanced technologies such as high throughput screening, combinational chemistry and genomic applications have been implemented to invent new antibacterial molecules to reverse antibiotic resistance [33]. An investigation conducted by Kim Lewis in 2001 lead to the key discovery of synergistic compounds of plant origin. His finding elucidated that a compound known as 5'-methoxyhydnocarpin-D isolated from the extracts of *Berberis fremontii* was able to potentiate the antibacterial action of berberine, inhibiting the activity of multidrug efflux pumps in Gram-positive and Gram-negative bacteria [34–36]. Novel approaches have been taken to combat antibiotic resistance such as the development of therapeutics based on anti-QS agents or bacterial quorum quenchers from natural products and bacterial vaccines. Unlike conventional treatments involving antibiotics, these novel therapies can be more potent and robust in combating advanced conditions of antibiotic resistance and bacterial virulence [37]. The implementation of bacterial vaccines is well evident in controlling diseases like tetanus, diphtheria, cholera, bacterial meningitis, typhoid fever and even anthrax, where measures have been taken to neutralize the outbreak of the disease in Swedish nature reserves in 2011 by vaccinating the resident animals against anthrax when treatment with penicillin was ineffective. Given this ideal, it has been predicted that such approaches will be nigh-impervious to resistance in bacterial populations and more robustly prevent the spread of infection [38, 39].

## Anthrax and the biological agent *Bacillus anthracis*

Anthrax is a serious enzootic infectious disease transmitted from infected livestock animals to humans. The biological agent responsible for causing anthrax is a Gram-positive endospore forming bacilliform bacterium known as *Bacillus anthracis*. Anthrax can be primarily acquired through direct contact and the consumption of contaminated meat. The most common forms of the disease under natural settings are cutaneous and gastrointestinal anthrax. Other but rare means of acquiring the disease include the inhalation of bacterial endospores that can result in pulmonary anthrax. The endospores remain dormant until being inhaled by a host and internalized, where they mature into toxin producing virulent bacterial cells in thoracic lymph nodes that cause acute and severe infection. The tactical delivery of concentrated endospores obtained from wild type *B. anthracis* is a strategy used in biological warfare and bio-terrorism. The disease is endemic to agricultural regions of southwestern and central Asia, Central and South America, southern sub-Saharan Africa, the Caribbean and Eastern Europe [40]. The largest agricultural outbreak of anthrax was reported from Zimbabwe, with cases of infection exceeding 10,000 between 1979 and 1985. It has been reported that nearly all cases of the infection occurred in Zimbabwe outbreak were cutaneous anthrax [41]. In the year 1979, about 79 cases related to inhalational anthrax were reported from Sverdlovsk region of Russia, in which 68 of those cases became ultimately fatal. The Sverdlovsk incident was the largest outbreak of human anthrax ever documented in history and is believed to have been caused due to an accident occurred in a Soviet military affiliated Microbiology facility that lead to the release of aerosolized anthrax spores [42]. The most recent incident related to anthrax bioterrorism was reported in 2001, in which concentrated spores of highly virulent *B. anthracis* were delivered using postal letters that resulted in about five fatalities among the 22 infected [43]. The disease incidence was significantly reduced during the twentieth century. Hence, anthrax continued to represent globally outside the United States, with an occurrence of approximately 2000 cases annually by the end of the twentieth century. A majority of these worldwide cases were associated with cutaneous anthrax [42]. The standard therapy for anthrax include with antibiotics like penicillin G procaine, doxycycline or ciprofloxacin being first-line treatment for the infection [44]. Newer anthrax therapeutic agents like monoclonal antibody based Anthim (obiltoxaximab) and the anthrax immune globulin based Anthrasil were also deployed solo or in combination with antibiotics to control the infection more effectively [41, 45]. In 2015, the Food and Drug Administration (FDA) approved BioThrax which is an immunologically active *B. anthracis* antigen vaccine against anthrax to prevent the disease. It is the only anthrax vaccine that has received FDA approval up to date [46]. The treatment of acute anthrax can be difficult due to the virulence properties of *B. anthracis*, in which the bacterium and its endospore are both encapsulated with a protective polysaccharide coating that allows immune evasion from phagocytes like macrophages. Bacterial exotoxins secreted by *B. anthracis* are known as edema toxin and lethal toxin which cause diarrhea and flu-like symptoms. The entry of these exotoxins into host cells and initial pathogenesis is facilitated by a major virulence factor present in *B. anthracis* known as the protective antigen. The polysaccharide capsule and other associated virulence factors are expressed by bacterial DNA plasmids, pXO1 and pXO2 present in *B. anthracis*. Although the bacterium can be eradicated by antibiotic agents, the toxins produced by the bacterium remain nonresponsive to antibiotic therapy. Hence, the CDC recommends the employment of a combined course of rapid antibiotic therapy involving two or three antibiotics along with anthrax anti-toxin therapy in order to prevent the accumulation of exotoxins in the body [47–49]. Antibiotic resistance in *B. anthracis* has been documented. One study showed that 11.5% out of 96 isolates of *B. anthracis* recovered from France between the time period of 1994 and 2000 indicated resistance amoxicillin and penicillin G. The same investigation revealed that all 96 isolates of *B. anthracis* tested were resistant to cotrimoxazole [50]. Although drug resistance mechanisms of *B. anthracis* has not yet been fully exploited, a study conducted by Price et al. showed that efflux-pump encoding bacterial genes gyrA, gyrB and parC can mediate cross-resistance to fluoroquinolone antibiotics like ciprofloxacin in *B. anthracis* [51]. Another study stated that *B. anthracis* consist of genes bla1 and bla2 that are capable of expressing beta-lactamases against β-lactam antibiotics [52]. Despite the treatment of anthrax infection with currently available antibiotics, the introduction of safer and more efficient chemotherapeutic options are required. Studies have demonstrated anti-*B. anthracis* activity of novel compounds extracted from medicinal plants and therefore, new insights involving the efficiency of plant-derived compounds and antibiotic combinations exhibiting anti-anthrax potential are needed to be addressed.

## Superbug bacteria

The continuous or inappropriate use of antibiotics has resulted in the development of extensive drug resistance in bacteria. Overtime, these organisms will progressively advance and evolve into superbug bacteria as an adaptive response to selective antibiotic pressure [53, 54]. The WHO has defined these bacteria as “Superbugs” due to the infections caused by these organisms are no longer treatable with existing antibiotic agents [55]. The CDC has categorized these organisms as urgent, serious, concerning and watch-list pathogenic threats. Antibiotic resistance is highly prevalent among Gram-positive bacteria, in which some of the well known examples of superbugs include methicillin-resistant *Staphylococcus aureus* (MRSA) and vancomycin-resistant enterococci (VRE). The United States national antimicrobial surveillance data has indicated the emergence more serious superbug infections associated with extended-spectrum beta-lactamases (ESBL) or carbapenemase producing organisms like *Klebsiella pneumoniae* and *Eschericia coli*, MDR pathogens like *Pseudomonas aeruginosa, Vibrio cholera* and *Clostridium difficile*, *Salmonella* spp. and drug-resistant *Mycobacterium tuberculosis*. However, some of the most worrisome threats up to date are associated with emerging superbugs like carbapenem-resistant *Acinetobacter* spp., particularly *Acinetobacter baumannii* [56].

### Methicillin-resistant *Staphylococcus aureus*

Methicillin-resistant *Staphylococcus aureus* (MRSA) are Gram-positive bacteria arranged in round-shaped clusters. They are among the most successive pathogens in the modern time period. *S*. *aureus* can exist as part of the human flora and can cause opportunistic infections. Being genetically diverse, these organisms can evolve into epidemic strains like MRSA [57, 58]. These MRSA are formidable clinical threats and are considered as archetypal hospital superbugs that are responsible for causing serious bloodborne infections like sepsis and infective endocarditis [59, 60]. According to CDC, more than 120,000 cases of hospitalizations reported in the United States from 1999–2000 were due to *S. aureus* associated infections. Among these cases MRSA accounts for 43.2% of infections in those hospitalized [61]. Antibiotic agents vancomycin or daptomycin are usually the first-line treatment for MRSA bacteraemia and related infections. However, in case of severe infection, combined therapy with flucloxacillin is given for more effective treatment. The primary reason for methicillin and other β-lactam-resistance in *S. aureus* is due to the expression of a foreign PBP known as PBP2a by the mecA gene present in them. PBP2a variant binds with β-lactam antibiotics with reduced avidity, which mediates resistance to this class of antibiotics. Lower affinity of PBP2a to β-lactam agents allows MRSA to replicate due to peptidoglycan synthesis taking place in the presence of β-lactams antibiotics that are capable of inactivating transpeptidase activity of PBPs. PBP2a is composed of a non-penicillin-binding protein and transpeptidase domain. Mutations in *S. aureus* associated genes like mprF, yycH, and dltA are also known for conferring cross resistance to daptomycin. *S. aureus* are also capable of acquiring genes that encode for antibiotic resistance from their predecessors. MRSA consist of a wide range of dynamic virulence factors that include immune-evasive bacterial surface factors (e.g. protein A and capsule) and tissue invasion promoting enzymes like hyaluronidase and toxins (e.g. leukocidins and haemolysins) for mediating pathogenesis. Two additional virulence factors known as Panton–Valentine leukocidin and arginine-catabolic mobile element have been discovered in a MRSA isolate called USA300, which facilitated the rapid spread of the strain by improving its adaptability to the pH of the human skin [62, 63]. Research has been undergoing in order to introduce newer and more effective therapies for MRSA infections such as the development of vaccines and potent natural products.

### Vancomycin-resistant enterococci

Vancomycin-resistant enterococci (VRE) are round-shaped Gram-positive bacteria that can cause serious MDR infections and persistent colonization in humans. Enterococci are opportunistic inhabitants that exist in the environment with an exceptional ability to adapt and evolve to transmit antibiotic-resistant determinants [64]. VRE can cause life-threatening infections in humans such as bloodstream infections like sepsis, endocarditis and pyelonephritis, in which most of these are nosocomial [65]. Tan et al. reported that a survey conducted from 2014 to 2016 showed that 523 out of 5,357 patients from health-care facilities in Singapore suffered from infections caused by VRE. An outbreak of VRE related infections has also been reported in 1997 from acute-care hospitals in the United Sates [64]. Linezolid is usually recommended as the first-line treatment for VRE and whereas, daptomycin, tigecycline and quinupristin-dalfopristin combined therapy are considered as last resort antibiotic agents for the management of enterococci related infections. The primary mechanism of drug resistance in enterococci involves the alteration of pathways associated with peptidoglycan synthesis, which specifically substitute D-Alanine-D-Alanine (D-Ala-D-Ala), to either D-Alanine-D-Serine (D-Ala-D-Ser) or D-Alanine-D-Lactate (D-Ala-D-Lac). These termini in VRE cell walls bind poorly with glycopeptide antibiotics like vancomycin [66]. A total of eight van gene clusters, vanA, vanB, vanD, vanE, vanG, vanL, vanM, and vanN are responsible for expressing elements required for antibiotic resistance in enterococci with vanA and vanB being the most abundant [67]. VanA is responsible for mediating the primary mechanism for antibiotic resistance in enterococci [68]. In addition to drug-resistance mechanisms, these enterococci consist of virulence factors like DNAse, caseinase and gelatinase to promote pathogenesis [69].

### Extended-spectrum beta-lactamases and carbapenemase producing *Klebsiella pneumoniae*

Extended-spectrum β-lactamases (ESBL) and carbapenemase producing *Klebsiella pneumoniae* are rod-shaped Gram-negative bacteria that cause high morbidity and mortality among hospitalized patients under intensive-care and neonatal intensive-care. These organisms are capable of producing carbapenemase against carbapenems and a rapidly evolving class of β-lactamase enzymes known as extended-spectrum β-lactamase, which have the ability to hydrolyze the β-lactam ring of a range of third/fourth-generation cephalosporin antibiotics and render them ineffective. EBSL and carbapenemase mediated drug resistance to numerous antibiotics make it challenging to treat infections caused by these organisms. *Klebsiella* spp. are ubiquitous in nature that belong to the family of bacteria called *Enterobacteriaceae*. These organisms exist in the natural environment and are a part of the human flora. *K. pneumoniae* are opportunistic pathogens, which have the ability to colonize the respiratory tract, gastrointestinal tract, genitourinary tract and eyes of those vulnerable [70]. Appropriate first-line treatment for EBSL producing *K. pneumoniae* include with antibiotics like amoxicillin/clavulanic acid, ceftriaxone, ciprofloxacin or cotrimoxazole) [71]. In case of carbapenemase producing *K. pneumoniae*, the treatment options include high-dose or combined antibiotic therapy with meropenem, tigecycline and/or colistin, gentamicin or fosfomycin depending on susceptibility [72]. Müller-Schulte et al. stated that 94% of infections reported from the University Teaching Hospital in Bouaké, West Africa from 2016–2017 were caused by EBSL producing *K. pneumonia* [73]. A survey conducted from 2014–2015 in long-term acute care hospitals based in the United States indicated that nearly 25% of infections caused in hospitalized patients were due to carbapenemase producing *K. pneumonia* [74]. Genes responsible for coding EBSL mediated antibiotic resistance in *K. pneumoniae* include blaSHV, CTX-M and TEM [75]. Navon-Venezia et al. indicated that plasmid genes blaVIM-1, blaOXA-48, blaVIM and blaNDM-1 are responsible for carbapenemase mediated drug resistance in *K. pneumoniae* [76]. Additionally, *K. pneumoniae* consist of virulence factors for facilitation pathogenesis such as capsular (K) antigen for evading phagocytosis, O antigen for invasion of host cells and siderophores like enterobactin and aerobactin for iron acquisition [77].

### Extended-spectrum beta-lactamases and carbapenemase producing *Eschericia coli*

ESBL and carbapenemase producing *Eschericia coli* are Gram-negative rod-shaped bacteria. These organisms are responsible for causing serious community and hospital-acquired infections worldwide, especially in places where inadequate hygienic practices are common and poor sanitation. *E. coli* is well known for causing gastroenteritis and infections associated with the urinary tract [78, 79]. In case of EBSL producing *E. coli*, the organism is responsible for causing bacteraemia in more than 5000 cases of hospitalized patients in the United Kingdom [80]. A prevalence survey conducted in a Spanish University Hospital indicated that 7.69% and 1.83% of admitted patients out of 10,643 suffered from infections associated with ESBL producing *E. coli* and carbapenemase producing *E. coli* respectively [81]. Antibiotics like colistin and carbapenems are usually the first-line treatment for ESBL producing *E. coli* [82]. Fritzenwanker et al. suggest that ertapenem infusion with meropenem or doripenem combine antibiotic therapy is given infections caused by carbapenemase producing *E. coli* [83]. A study conducted by Overdevest et al. showed that ESBL producing *E. coli* harbored plasmid genes like blaCTX-M-1 and blaTEM-52 [84]. Shin et al. detected blaNDM–5 gene in high level carbapenemase-resistant *E. coli* [85]. Aside from antibiotic resistance, *E. coli* has a number of virulence factors for mediating pathogenesis like heat-labile toxin, heat-stable toxin, enterohaemolysin, shiga-like toxin, enteroaggregative heat-stable, enterotoxin, haemolysin, cytotoxic necrotizing factor, uropathogenic specific protein and invasin for host cell invasion and K1-capsule and intimin for immune evasion and cellular attachment [86, 87].

### Multidrug-resistant *Pseudomonas aeruginosa*

*Pseudomonas aeruginosa* are Gram-negative bacteria arranged in rods or bacilli. These organisms can be found in the environment (e.g. soil and water) and are known for causing blood borne infections and pneumonia in humans under opportunistic conditions [88]. *P. aeruginosa* are also associated with hospital-acquired infections, in which MDR *P. aeruginosa* is responsible for causing 32,600 infections among patients who were hospitalized and 2700 fatalities in the United States in 2017 [89]. Monotherapy and combined therapy with antibiotic agents like ceftolozane-tazobactam, ceftazidime-avibactam, cefiderocol and imipenem-relebactam/cilastatin are used for the treatment of infections caused by MDR strains of *P. aeruginosa* [90]. The most common mechanism of antibiotic resistance in *P. aeruginosa* is associated with the overproduction of drug efflux pump systems like MexAB-OprM, MexEF-OprN, MexXY-OprM and MexCD-OprJ induced by mex gene mutations. These multi-drug efflux pumps function as antibiotic molecule extruders. Apart from efflux pumps, these organisms also consist of genes like AmpC that code for the production of β-lactamases and OprD for encoding alterations in type II topoisomerases (DNA gyrase) to mediate resistance against fluoroquinolone and carbapenem antibiotics [91]. *P. aeruginosa* also expresses a number of virulence factors such as protease A, exotoxins, phospholipase C and cytotoxins for host cell invasion and pyoverdine and QS system regulatory proteins essential for the formation of biofilms, that plays a vital role in host immune evasion and antibiotic resistance [92].

### Multidrug-resistant *Vibrio cholera*

*Vibrio cholera* are comma-shape Gram-negative bacteria well known for causing the severe water-borne acute, diarrheal illness cholera. These organisms have become a major threat to public health, particularly in the developing world [93]. According to recent reports, the *V. cholerae* O1 El Tor variant is responsible for causing cholera outbreaks worldwide [94]. The transmission of *V. cholerae* generally occurs via the faecal-oral route by ingesting contaminated water and food [95]. Typically, doxycycline is used as the first-line treatment for cholera infection caused by *V. cholera* [96]. According to CDC, *V. cholerae* colonizes the small intestine to cause cholera and an estimation of 2.9 million cases and 95,000 fatalities occur annually worldwide [97]. WHO estimates that 1.3 to 4 million cases of pathogenic infections and 21,000–143,000 fatalities reported across the globe are due to cholera [98]. It has been reported that *V. cholerae* have caused seven pandemics related to cholera in different countries [99]. MDR *V. cholerae* has a number of antibiotic resistance mechanisms such as active antibiotic molecule efflux, reduced cell wall permeability to antibiotics, alteration of binding targets sites for antibiotics via undergoing post-transcriptional or translational modifications (e.g. mutations in topoisomerase and DNA gyrase) and hydrolysis or chemo modification of antibiotic agents. These resistance mechanisms are expressed by genes like blaNDM-1, blaDHA-1, carR, ant 3’, tet(M), tetD, foIP, qacEΔ1, mph2, mel, armA, rmtB, rmtC, rmtF, aphA1, arr2, bcr, mphRK, mrx, blaP, vigA, blaCTX-M, sh ble, floR, cat, aacA, aphD, tetG, aac-Ib, qnrVC3, ereA2, bla, strA, strB, sul2, mdtH, rpsl, dfrA, dhfrII, aad3’ and mph in MDR *V. cholerae* [100]. Major virulence factors necessary for mediating pathogenesis and host cell invasion in *V. cholerae* include the cholera, toxin-coregulated pilus and O antigen [101].

### Multidrug-resistant *Clostridioides difficile*

*Clostridioides difficile* are Gram-positive spore-forming bacteria that are ranged in rods or bacilli. These organisms are responsible for causing colitis and nosocomial diarrhea. *C. difficile* are opportunistic pathogens, in which they colonize the small intestine when the gut microbiota is disrupted as a result of antibiotic misuse [102]. Metronidazole is the first-line treatment mild to moderate infection, whereas advanced forms of infection are treated with vancomycin and fidaxomicin monotherapy or combined therapy. *C. difficile* infection is more prevalent among the elderly who have prescribed antibiotics for other conditions [103, 104]. CDC reports that 223,900 patients admitted to hospitals and 12,800 fatalities in the United States in 2017 were associated with *C. difficile* infections [105]. Data obtained from the French National Uniform Hospital in 2016 indicated that 3.6 cases per 10,000 acute care patient days account for infections caused by *C. difficile* [106]. MDR strains of *C. difficile* consist of genes like gyrA for mediating moxifloxacin and rpoB for mediating resistance against rifampicin. Moreover, *C. difficile* consist of genes associated with tetracycline resistance like tetM and genes that code for aminoglycoside-modifying enzymes like aac(6′)-aph(2″) and aadE. *C. difficile* also expresses genes like mef(A)-msr(D), ermG, and vat for coding resistance to antibiotics lincosamide, streptogramins and macrolide. A mutation associated with Cys721Ser PBP in *C. difficile* has been speculated to contribute resistance towards imipenem [107]. The bacterial exotoxins TcdA, TcdB and binary toxin are the common virulence factors associated with *C. difficile* for host invasion and promoting pathogenesis [108].

### Drug-resistant *Mycobacterium tuberculosis*

*Mycobacterium tuberculosis* are acid-fast bacilli that has been categorized under serious threats by the CDC. These organisms are well known for causing the highly infectious lung associated disease named tuberculosis (TB) [109]. The transmission of *M. tuberculosis* occurs via droplet nuclei from an infected person [110]. TB caused by MDR, XDR or pandrug-resistant (PDR) strains of *M. tuberculosis* poses a serious threat to the public health worldwide, in which the disease has claimed the lives of 1.3 million and about 8.6 million cases of the infection has been reported in 2012 [111]. The WHO in 2016 estimated that there were 600,000 cases of TB and 240,000 fatalities attributed to the disease have been reported [112]. FDA approved first-line treatment anti-TB agents include isoniazid, rifampin, ethambutol, pyrazinamide [113]. In case of MDR or XDR TB, anti-TB drugs like pretomanid in combination with linezolid and bedaquiline are given [114]. Bacillus Calmette–Guérin immunotherapy is generally used to prevent TB, which uses a live-attenuated vaccine derived from *Mycobacterium bovis* to immunize against the TB infection [115]. *M. tuberculosis* consists of several genes capable of mediating antibiotic resistance via drug target modulation. These include katG, inhA, ndh and ahpC targeted against isoniazid, rpoB against rifampicin, pncA and rspA targeted to counter pyrazinamide, embCAB and embR that modulates binding sites of ethambutol, rpsL, rrs and gidB targeted against streptomycin, rrs and eis modulates binding sites of amikacin/kanamycin, ethA, inhA, ethR, ndh and mshA to counter ethionamide and gyrA and gyrB modifies DNA gyrase against fluoroquinolones [116]. The major virulence factors for promoting pathogenesis in *M. tuberculosis* include phthiocerol dimycocerosate for host cell invasion and phenolic glycolipids involved in the evasion of host immune responses and inducing macrophage toxicity [117].

### Multidrug-resistant *Salmonella* spp.

*Salmonella* spp. are Gram-negative zoonotic disease causing enteric bacteria. Over 2600 serotypes of *Salmonella* have been identified, which are responsible for causing gastrointestinal diseases such as food poisoning. Depending on the nature of symptoms, *Salmonella* infections can be classified as non-typhoidal, paratyphoidal and typhoidal, in which both paratyphoidal and typhoidal *Salmonella* causes high fever (typhoid fever). Most cases of foodborne infections have been found to be associated with *Salmonella enterica* serovar Enteritidis [118–120]. The transmission of *Salmonella* occurs via the fecal–oral route by ingesting contaminated food [121]. Generally, the first-line treatment for *Salmonella* infections include with antibiotics like fluoroquinolones for adults and azithromycin for children pediatric patients. Alternatively, ceftriaxone can also be used as a first-line antibiotic therapy for *Salmonella* [122]. The FDA has approved *Salmonella* vaccines such as Vi bacterial polysaccharide (Vi antigen) under the brand name Typhim Vi and Vivotif that uses a live-attenuated ty21a strain via oral administration for the immunization against typhoid fever [123]. According to CDC, about 1.35 million community infections, 26,500 cases of hospitalizations, and 420 fatalities in the United States annually are associated with *Salmonella* [124]. Zhang et al. stated that about 70–80% of outbreaks of foodborne illnesses in China are caused by *Salmonella* bacteria [125]. A global study conducted by WHO indicated that 21,650,974 cases of *Salmonella* infections caused typhoid fever, which resulted in death among 216,510 of the infected and 5,412,744 cases suffered from paratyphoid fever [126]. Zhang et al. revealed that mutations in AcrAB gene mediate antibiotic resistance in *Salmonella* by overexpressing AcrAB-TolC bacterial efflux pumps. The findings of the same study showed that genetic mutations in GyrA and GyrB can alter DNA Gyrase in *Salmonella*, which are known for facilitating the development of resisatnce against ciprofloxacin. Another study revealed that aac(6′)-I gene is frequently associated with aminoglycoside resistance. Moreover, the β-lactamase producing blaCMY-2 gene and tetR** gene that encodes for tetracycline resistance were abundantly present in *Salmonella* [127]. There are a number of associated with *Salmonella* such as the Vi capsular antigen, somatic O antigen, H antigen (flagella), fimbriae and type III secretion systems that include Salmonella pathogenicity island 1 (SPI-1), SPI-2, which promote host cell invasion and pathogenesis [128, 129]. The cytolethal distending toxin in *Salmonella* has been found to cause typhoid fever among the infected. Other salmonellosis mediating toxins like pertussis-like toxin A and pertussis-like toxin B were also have been found to be expressed by these organisms [130].

### *Acinetobacter baumannii*

*Acinetobacter baumannii* is accountable for causing most community and hospital-acquired infections. Overtime, these organisms can evolve into XDR or PDR superbugs as a result of continuous selective pressure and rendering the majority or all existing antibiotics ineffective. The CDC has alerted and listed *A. baumannii* as an organism that needs to be considered as an urgent threat [56, 131, 132]. *A. baumannii* is a coccobacillus Gram-negative bacterium that is known for colonizing the gastrointestinal, respiratory tract and the oral cavity of humans. It is also recognized as a formidable opportunistic pathogen that causes many forms of severe recalcitrant infections. *A. baumannii* infections are resulted frequently due to wound contamination [133]. However, it is a clinically dominant bacterial species that has a pronounced tendency to cause healthcare-associated nosocomial infections [134, 135]. *A. baumannii* has been listed under ESKAPE pathogens and is the most aggravating member of the Acb-complex (*A. calcoaceticus, A. baumannii* and Acinetobacter genomic species 13TU) that have been found to show high resistance to antibiotic agents which increases the risk of mortality among hospitalized patients under intensive care [133, 134, 136–140]. Although infections caused by this bacterium were able to keep under control in early 1970s, *A. baumannii* lately re-emerged as MDR and XDR strains with marked resistance to most antibiotics like gentamicin, nalidixic acid, minocycline, carbenicillin, and ampicillin. The bacterium exhibited resistance to a majority of antibiotics during early 1990s and by late 1990s, the only treatment of choice was carbapenems in combination with rifampicin [141, 142]. Presently, infections caused by MDR and XDR strains of *A. baumannii* were being treated with antibiotics like polymyxin B, colistin and tigecycline. However, more new strains of *A. baumannii* have been frequently reported that can exhibit resistance to the aforementioned antibiotics [143, 144]. The extensive resistance to antibiotics in *A. baumannii* is primarily due to the prevalence of adaptive multidrug efflux pumps like adeA, adeB, adeC, adeDE, adeABC, adeFGH, adeXYZ and adeIJK in the bacterium [145]. *A. baumannii* harbors a multitude of virulence factors for facilitating host cell invasion and pathogenesis such as the biofilm promoter outer membrane protein A, surface antigen 1, lipid A, phospholipase, secretion systems (type 1, type 2, type 4, type 5, type 6 and type V), siderophores for iron acquisition, binding domains for the acquisition of zinc and magnesium and drug resistance promoting QS systems [146]. These rapid mutations in *A. baumannii* make it one of the most challenging biological factors to human health and public health-care systems. The recent emergence of pandrug-resistant superbugs like *A. baumannii* has indicated an urgent necessity for the discovery of novel antibacterial agents and chemotherapeutic strategies [137, 139].

## Plant-derived compounds as antibacterial agents

**Fig. 1:**
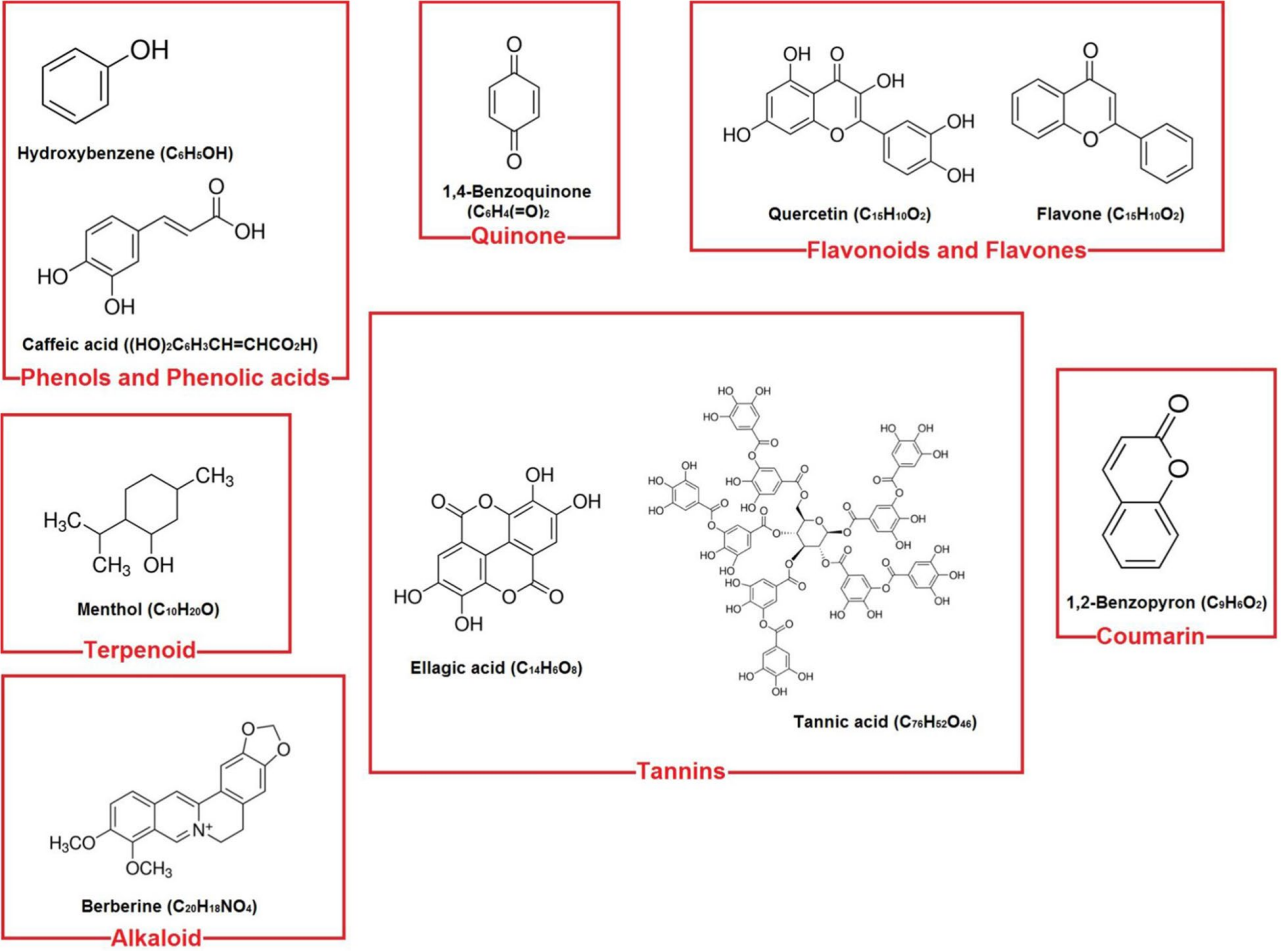
Chemical structures of plant compounds generally isolated from plants

Plants are natural factories capable of producing a series of different phytochemical compounds. These compounds were produced in response to adverse biotic and abiotic environmental conditions. Phytoconstituents have a major impact on other plants, animals and microorganisms in their immediate environment that surrounds them [147]. Plant-derived constituents are biologically active organic compounds and are generally defined as secondary metabolites. These secondary metabolites are structurally diverse compounds that are classified into three primary groups as phenolic compounds (phenolic acids, simple phenols, flavonoids, quinones, coumarins and tannins), alkaloids and terpenes (Fig. 1). These compounds can be isolated from crude extracts and essential oils of plants. Complex mixtures of phytochemicals are represented in crude extracts that contain primary and secondary metabolites of different classes, chemical and biosynthetic. These compounds share some of the common mutual characteristics, such as volatility and/or polarity. Since antiquity, extracts obtained from medicinal plants have been known to have broad-spectrum antimicrobial activities and have been frequently studied and reviewed. Their profound antibacterial activity is generally recognized as a safe substance and, with the minimal risk of developing bacterial resistance, has qualified them as suitable sources for the development of new antibacterial agents [148, 149].

## Mechanism of action of plant-derived antibacterial compounds

The efficiency of antibacterial compounds derived from plants depends on several factors, such as features of test microorganism (type, species and strain), botanical source and composition of the bioactive phytochemical compounds, as well as the stage of development, time of harvesting of the plant material and most importantly, the method of plant extraction. Due to the complex nature of the compounds present in crude extracts of plants, they can exhibit multiple mechanisms of action on bacteria. These include the suspension of bacterial growth, function or viability, targeting bacteria virulence factors, potentiating the effectiveness of antibiotics as agents that modify bacterial resistance. Similar to antibiotic agents, these phytochemicals can inhibit the growth and replication of bacteria, disrupting the structure and function of the bacterial cell membrane [150], interrupting the synthesis of nucleic acids such as DNA or RNA [151], disrupting the intermediary metabolism [152], and the coagulatory induction of bacterial cytoplasmic constituents [153].

Several studies have been conducted to understand and illustrate the antibacterial action of phenolic compounds such as flavonoids, coumarins and tannins. Flavonoids are a diverse group of polyphenolic compounds that have the ability to inhibit the activities of DNA gyrase and DNA topoisomerase, energy metabolism mediated by NADH-cytochrome C reductase or inhibition of ATP synthase and the interruption of components involved in the synthesis of the cell wall and cell membrane [154]. Possible targets of quinones include peptidoglycan from the bacterial cell wall and enzymes associated with the cell membrane [151]. It is known that tannins cause the destabilization of the cell membrane and alterations in metabolic pathways and inactivation of membrane-bound proteins [155]. Phytochemicals like coumarins mediate the delay in bacterial cell respiration. Terpenes disrupt the bacterial cell membrane due to their lipophilic nature. Alkaloids are some of the most widely studied plant-derived compounds which can intercalate with bacterial DNA and enzymes associated with nucleic acids as esterase, DNA or RNA polymerases [156]. Examples of antibacterial mechanisms of action of plant secondary metabolites against CDC classified bacterial superbugs and anthrax biological agent *B. anthracis* are elucidated in Table 1.

**Table 1:** Representative studies on phytochemical constituents generally used for antibacterial activity against *B. anthracis* and superbug bacteria

Phytochemical		Bacteria	Mechanism of action	MIC of plant compound	References
Class	Compound				
Flavonoids	Glabrol	MRSA	Bacterial cell membrane disruption and dissipation of proton motive force	–	[244]
	Datiscetin,	*S. aureus*	Interfere with the synthesis of DNA and ribosomal RNA synthesis	–	[245]
	Morin,				
	Quercetagetin,				
	Robinetin,				
	Myricetin,				
	Galangin,				
	Kaempferol,				
	Fisetin,				
	Quercetin,				
	Dihydrorbinetin				
	Norwogonin	*A. baumannii*	–	128 μg/mL	[140, 246, 247]
	Baicalein		–	–	
	Baicalin		–	–	
	Luteolin		Protein leakage from bacterial cell	–	[260]
	Isoorientin				
	Epicatechin gallate		–	–	[231, 248]
Quinones	Haloemodins	MRSA and VRE	Inhibition of DNA gyrase	–	[224]
	Anthraquinone	*B. anthracis*	–	130 μg/μL	[302]
Alkaloids	Tomatidine	*Bacillus* sp*.* *Staphylococcus* sp., *Listeria* sp*.*	Inhibition of ATP synthase activity	– –	[15, 249]
	Berberine	*A. baumannii, E. faecalis, S. aureus*	–	–	[36, 139]
		*S. pyogenes*	–	30 μg/mL	
Organosulfur compounds	Allicin	*S. epidermidis, S. agalactiae*	Inhibition of Sulfhydryl-dependent enzyme, Inhibition of DNA and protein synthesis	–	[250]
	Ajoene	*Streproproteus* sp., *Staphylococcus* sp.	Sulfhydryl-dependent enzyme inhibitor	–	[251]
	Allyl methyl trisulfide	*A. baumannii*	–	3120 μg/mL	[140]
Phenols	p-Coumaric acid	*O. oeni, L. hilgardii*	Damage to the cytoplasmic membrane	–	[252]
		*S. aureus, S. pyogenes, B. cereus, B. subtilis*	–	–	[140, 253]
	3-p-Trans-coumaroyl-2-hydroxyquinic acid	*S. aureus*	–	–	[254]
Phenols	Thymol	*A. baumannii*	–	–	[140, 255]
	Epigallocatechin gallate	*A. baumannii*	–	312–625 μg/mL	[140, 164, 170, 256]
		*S. aureus*	–	100 μg/mL	
	Theaflavin	*A. baumannii*	–	256–512 μg/mL	[40, 248, 257]
	Paeonol	*A. baumannii, E. faecalis, S. aureus*	–	–	[244, 247]
	Honokiol,	*A. baumannii*	–	–	[244, 259]
	Magnolol		–	–	
	Sugiol	*S. aureus*	–	–	[244]
	Rosmarinic acid	*A. baumannii*	Protein leakage from bacterial cell	1000 μg/mL	[260]
	Sophoraflavanone B	MRSA	Direct interaction with peptidoglycan	–	[261]
	Naringenin, Eriodictyol, Taxifolin	*E. faecalis*	β-Ketoacyl acyl carrier protein synthase (KAS) III	–	[262]
Phenols	Curcumin	*S. aureus*	Causes leakage of constitutes from cell membrane	–	[263, 264]
		*A. baumannii*	–	4 μg/mL	
Coumarin	Aegelinol,	*S. aureus*	DNA gyrase inhibitor	–	[265]
	Agasyllin		–	–	
	4′-senecioiloxyosthol,	*B. subtilis*	–	–	[250]
	Osthole	*B. subtilis, S. aureus*	–	–	
	Asphodelin A 4′-O-β-D-glucoside,	*S. aureus*	–	–	[266]
	Asphodelin A		–		
Terpene	Farnesol	*E. faecium*	Cell membrane disturbance	–	[244]
	Nerolidol Plaunotol	*S. aureus*		–	[258]
	Oleanolic acid	*A. baumannii*	Protein leakage from bacterial cell	–	[141]
	Ursolic acid			–	
	Dehydroabietic acid	*B. anthracis*	Inhibits the cellular cytoplasmic entry of anthrax toxin	–	[267, 303, 304]
	Celastrol			–	
	Toosendanin			–	
Tannins	(4R)-(−)-carvone	*L. monocytogenes*	–	–	
	(4S)-( +)-carvone	MRSA, MSSA	Cell membrane disturbance	310 μg/mL	[138, 268, 269]
	Carvacrol	*A. baumannii*	–		[140]
	Eugenol	*A. baumannii*		1250 μg/mL	[140, 270]
	Cinnamaldehyde	*S. aureus*	Destabilization of plasma membranes, inhibition of metabolites and bacterial enzymes and deprivation of substrates needed for bacterial growth	–	[145]
	Ellagitannins	*L. monocytogenes*		–	[30, 271]
		*S. aureus*		–	[151, 271]
	Ellagic acid	*A. baumannii*	–	250 μg/mL	[140, 246]
		*B. cereus, S. aureus*	–	–	[145]
	Tannic acid	*S. aureus*	Cell membrane disturbance	–	[30, 271–276]
	Gallotannins				
	Procyanidins	*S. aureus,* *B. subtilis*		–	
	Terchebulin	*A. baumannii*	–	500 μg/mL	[140, 246]
	Chebulagic acid		–	1000 μg/mL	
	Chebulinic acid		–	62.5 μg/mL	
	Corilagin		–	1000 μg/mL	
	Prodelphinidins	*S. aureus* *B. subtilis*	Cell membrane disturbance	–	[30, 275, 276]
	Prorobinetinidins			–	
	Profisetinidins	*B. subtilis*	–	–	[272, 276]
	Hexahydroxydiphenoyl ester vescalagin	*A. baumannii*	–	–	[140, 277, 278]
		*S. aureus*	–	62 μg/mL	
		*B.cereus*	–	–	
	Trans-cinnamaldehyde	*A. baumannii*	–	310 μg/mL	[246, 279]
	Lyoniresinol-3 alpha-O-beta-D-glucopyranoside	*A. baumannii*	–	–	[140, 247]
		*E. faecalis*	–	–	
		*S. aureus*	–	–	
	Furanosesquiterpenes	*A. baumannii*	–	2500 μg/mL	[140, 280]
	4-cymene	*S. aureus*	–	1000 μg/mL	[132, 281]

## Methods for evaluating the antibiotic synergistic activity of plant-derived compounds

### Growth inhibitory indices

The agar diffusion assay based synergistic activity of antibiotic-plant extract combinations can be evaluated using the application of the growth inhibitory indices (GIIs), calculated according to the formula below:

$$\rm{GIIs }= \frac{\rm{Inhibiton \, zone \, diameter \, of \, antibiotic }+\rm{ plant \, extract \, combined \, disk}}{\rm{Inhibition \, zone  \, diameter \, of \, plant \, extract \, and \, antibiotic \, in \, individual \, action}}$$

The GIIs value > 1 will be considered as synergistic, 1 as additive, and < 1 as antagonistic [157].

### Fractional inhibitory concentration index

The fractional inhibitory concentration index (FICI) is used for the evaluation of synergism between two antimicrobial compounds in micro/macrobroth dilution assays. The FICI of the antibiotic-plant extract combination agent can be estimated using the standard formula shown below:

$${\rm{FIC}}_{\rm{Plant \, extract}/\rm{compound }}= \frac{\rm{MIC  \, of \,  plant  \, extract}/\rm{compound  \, in \,  combination \,  with  \, antibiotic}}{\rm{MIC \,  of \,  plant  \, extract}/\rm{compound}}$$

$${\rm{FIC}}_{\rm{Antibiotic }}= \frac{\rm{MIC \,  of \,  plant \, extract}/\rm{compound \,  in  \, combination \,  with \,  antibiotic }}{\rm{MIC \,  of \,  antibiotic}}$$

$${\rm{FIC  \, index}}_{\rm{Plant \,  and \,  antibiotic \,  combination}}= {\rm{FIC}}_{\rm{Plant \,  extract}/\rm{compound }}+ {\rm{FIC}}_{\rm{Antibiotic}}$$

FIC index ≤ 0.5 will be considered as synergistic, > 0.5 but < 1 as partially synergistic, additive when = 1, indifferent when > 1 but < 4 and ≥ 4 as antagonistic [158].

## Synergistic interaction between phytoextracts and antibiotics

Synergism between plant-derived compounds and existing antibiotic agents is an effective and an efficient way to manage the development of bacterial multi-drug resistance [159, 160]. Several studies have shown the significance of this type of synergistic interaction in the discovery of novel antibacterial agents. Phytochemicals are cable of interacting with synthetic antibiotic agents. This phytochemical and antibiotic interaction has been classified as antagonistic, additive or synergistic. The term antagonistic is given when a plant-derived compound reduces the effectiveness of an antibiotic agent against a certain type of bacteria, whereas the terms additive and synergistic are assigned to compounds that can enhance the antibacterial activity of the antibiotic [161]. An additive effect is usually considered as the baseline effect for detecting synergy in antimicrobial assays, in which such effect can be theoretically expected from a combination of multiple antimicrobial agents when the synergistic effect is absent. Synergistic effect can be defined as a combined effect that is significantly greater than the additive effect. A plant extract/compound fused with an antibiotic agent can be considered as a synergistic product when their combined action is superior to that of their individual antibacterial activity [162]. The distinctive action of phytochemical-antibiotic synergism is the ability to overcome antimicrobial resistance. Besides reducing antibiotic resistance, another advantage of this type of synergism is that it can reduce the minimum inhibitory concentration of an antibiotic agent, which also lower the dose needed for its effect to take place and mitigation of possible adverse effects [9, 163].

**Fig. 2:**
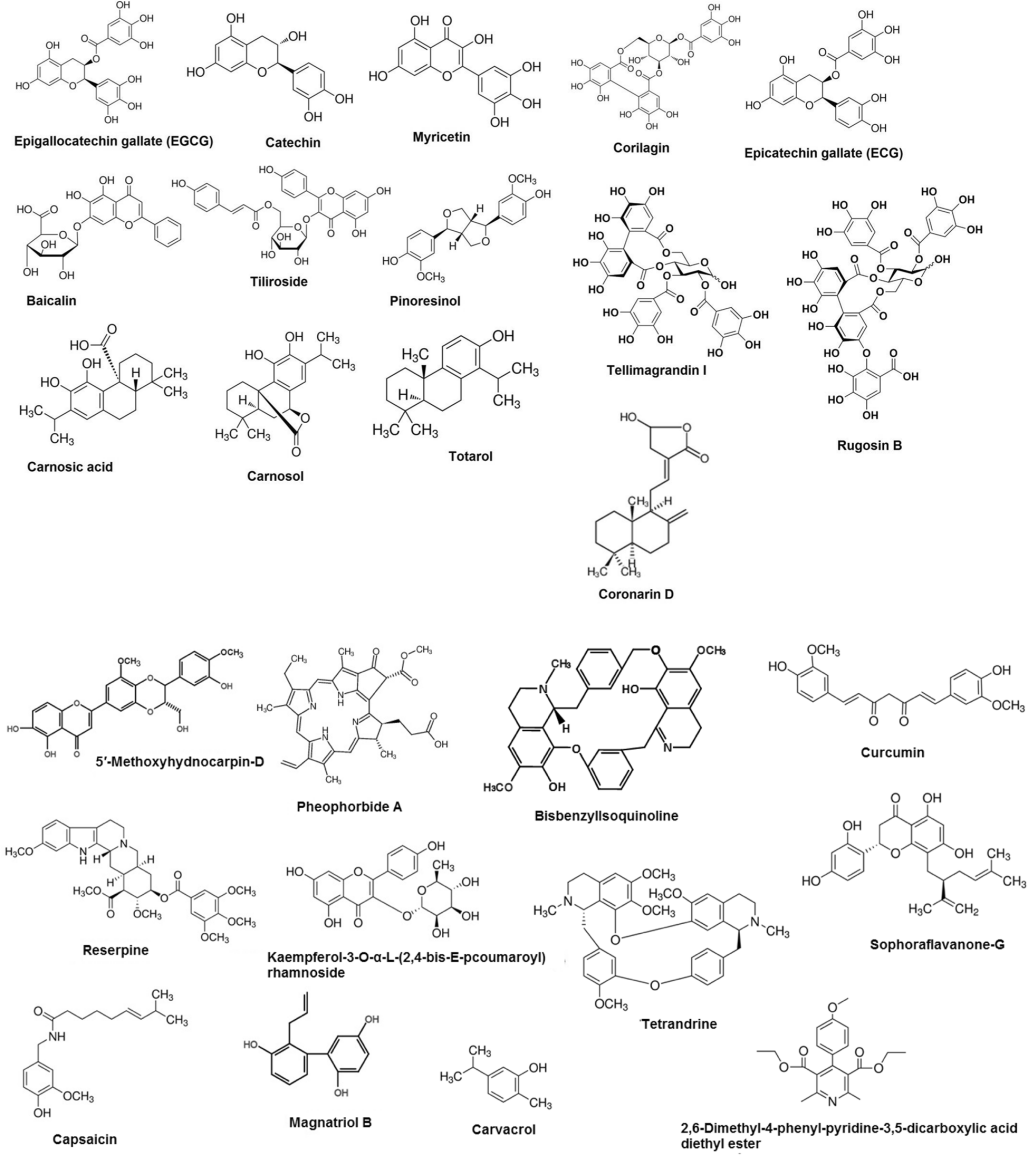

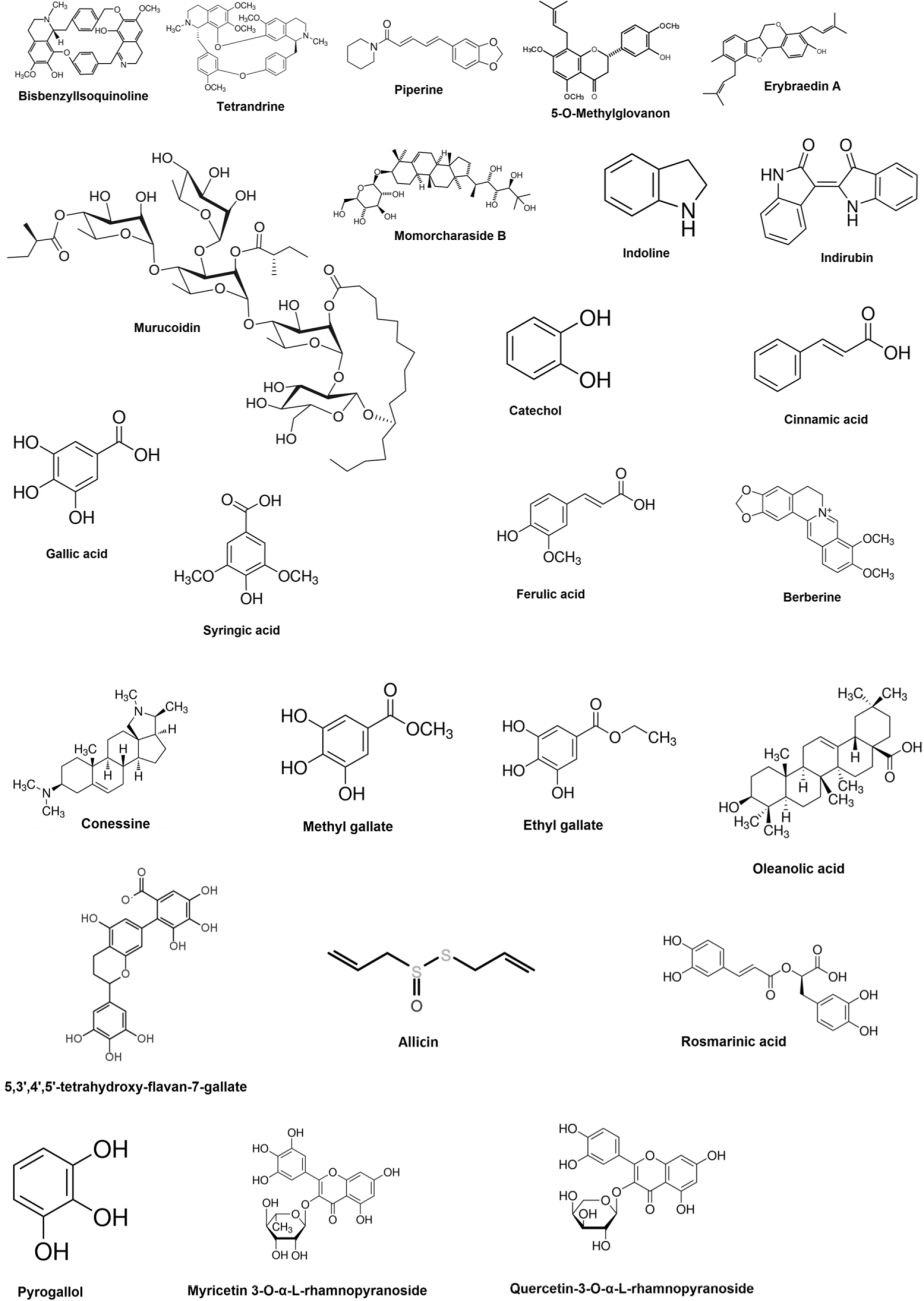

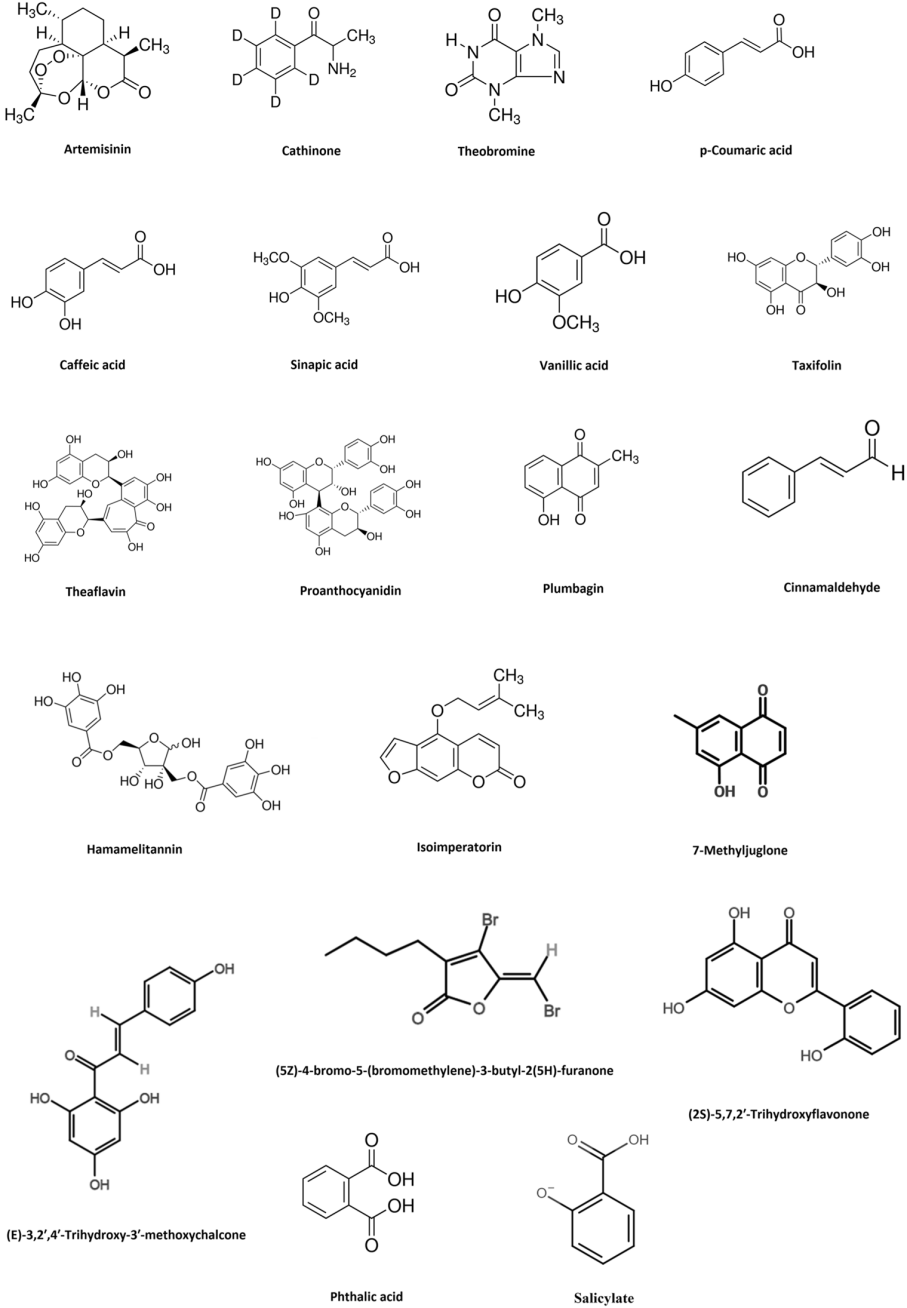
Chemical structures of isolated plant compounds studied for antibiotic synergistic activity

A recent study indicated that epigallocatechin gallate isolated from *Camellia sinensis*, which is a variant of green tea was able to potentiate the antibacterial action of sulfamylon (mafenide acetate) against a clinical isolate of *A. baumannii* [164]. Another study showed that phytoextracts obtained from plants like *Alstonia scholaris*, *Adenium obesum*, *Cerbera odollam*, *Cerbera manghus*, *Nerium oleander*, *Holarrhena antidysenterica*, *Plumeria obtuse*, *Wrightia pubescens*, *Thevetia peruviana*, *Punica granatum*, *Terminalia bellirica*, *Quisqualis indica*, *Terminalia* sp. and *Terminalia chebula* were able to synergistically potentiate the activity of seven antibiotic agents like cephazolin, rifampicin, meropenem, gentamicin, erythromycin, streptomycin, fusidic acid and novobiocin against *A. baumannii* ATCC 19606 [165–168]. Knezevic et al. reported that plant extracts obtained from *Eucalyptus camaldulensis* showed ciprofloxacin, polymyxin B and gentamicin potentiating effect when used against different strains of *A. baumannii* [169]. Isolated phytochemical compounds like tannic acid, catechol, cinnamic acid, ellagic acid, ferulic acid, gallic acid and syringic acid have exhibited novobiocin potentiating ability against *A. baumannii* JVC1053 [170]. A study indicates that extracts of *Levisticum officinale* was able to synergistically enhance the antibacterial action of cipofloxacin against MDR *A. baumannii* NCTC 13305 [171]. An investigation conducted by Mandal et al. showed that ethanolic extracts of *Ocimum sanctum* potentiated the antibacterial action of trimethoprim and chloramphenicol against *S. typhi* with highest GIIs ranging from 1.2 to 1.3 [157]. Plant extracts obtained from *Peganum harmala* L, *Cassis italic Mill*, *Carthamus tinctorius* have exhibited the antibiotic potentiating ability of ampicillin, cefotaxime, vancomycin, chloramphenicol, tetracyclines against *A. baumannii* with a FICI of 0.5 [172, 173]. Antibiotics like cefotaxime, tetracyclines, vancomycin, ampicillin and chloramphenicol showed enhanced antibacterial activity against clinical isolates of *A. baumannii* when used in conjunction with phytoextracts of *Terminalia chebula* and *Senna italica Mill* [167, 174]. Liu et al. revealed that isolated compounds present in *Pithecellobium clypearia* mediated synergistic antibacterial activity against *A. baumannii* when used in combination with imipenem, cefoperazone, ceftazidime, levofloxacin, amikacin, tetracycline and polymyxin B sulfate [175]. A study indicates that the isolated phytochemical compound known as berberine exhibited antibiotic potentiating ability of ciprofloxacin and Imipenem against *A. baumannii* [176]. Allicin (Fig. 2) isolated from *Allium sativum* (garlic) indicated FICIs of 0.5 and 0.38 when used in combination with cefazolin and oxacillin respectively against *S. aureus* and FICIs of 0.25 for both antibiotics when used in combination against *S. epidermidis* [158]. Ekambaram et al. showed that rosmarinic acid (Fig. 2) was able to synergistically potentiate the antibacterial activity of vancomycin, amoxicillin and ofloxacin when used in combination against *S. aureus* and MRSA with FICIs of 0.5 for each combination [177]. One study indicated that oleanolic acid (Fig. 2) was able to enhance the antibacterial action of kanamycin and gentamicin against *A. baumannii*. A FICI of 0.313 for gentamicin and 0.375 for kanamycin was indicated when combined with oleanolic acid [178]. Another study showed the antibiotic potentiating ability of *Zingiber cassumunar* extracts when used in combination with a range of broad-spectrum antibiotic agents like trimethoprim-sulfamethoxazole, amoxicillin, amoxicillin-clavulanic acid, piperacillin-tazobactam, ceftazidime, cefepime, ceftriaxone, imipenem, cefotaxime, meropenem, ertapenem, tetracycline, gentamicin, amikacin, gentamicin, doxycycline, ciprofloxacin and levofloxacin against an XDR strain of *A. baumannii* [179]. Isolated compounds from plants taken for the investigation of antibiotic synergism include bisbenzylisoquinoline, tetrandrine, carvacrol, curcumin, murucoidin, catechol, cinnamic acid, ferulic acid, gallic acid, syringic acid, berberine, methyl gallate, ethyl gallate, pyrogallol, myricetin-3-O-α-L-rhamnopyranoside, quercetin-3-o-α-l-rhamnopyranoside, 5,3',4',5'-tetrahydroxy-flavan-7-gallate, ellagic acid, (E)-3,2',4'-trihydroxy-3'-methoxychalcone, (2S)-5,7,2'-trihydroxyflavonone, 7-methyljuglone, isoimperatorin and tannic acid (Table 2 and Figs. 1, 2). A study indicate that crude seed and seed oil extracts of *Jatropha curcas* showed synergistic activity against both MDR and clinical strains of *E. coli*, *P. monteilii*, *P. aeruginosa,* MRSA, *E. faecalis*, MDR *A. baumannii* and *P. chlororaphis* when used in combination with cephazolin, rifampicin, fusidic acid ciprofloxacin, cefotaxime, rifampicin, moxifloxacin and ofloxacin. The seed oil extract of *J. curcas* combined with cefotaxime showed the best synergism in the study with an FICI of 0.005 against the clinical isolate of *P. aeruginosa* [180]*.* An investigation revealed that theaflavin (Fig. 2), an antioxidant polyphenol compound present in black tea was able to potentiate the antibacterial action of ampicillin with a FICI of 0.35 when tested against *S. maltophilia* [181]*.* Seed and root extracts obtained from *Peganum harmala* L. synergistically potentiated the antibacterial action of novobiocin and carbenicillin against *B. anthracis* isolated from a clinical specimen [182]. Moreover, a study conducted by Kouitcheu et al. showed that extracts obtained from *Cylicodiscus gabunensis, Picralima nitida, Cassia arereh* and *Trichilia emetic* interacted synergistically with gentamicin, erythromycin and kanamycin against *V. cholera*. These extracts were able to reduce the MICs of the selected antibiotic by 2 to 16 fold for *V. cholera* [183]. The hydro-alcholic extracts of *Oliveria decumbens* showed a synergistic effect when used in combination with tetracycline, oxacillin and doxycycline against *Brucella melitensis*. According to the investigation, *O. decumbens*-doxycycline combination indicated the highest anti-*Brucella* synergistic activity with a zone of inhibition that is 9 mm larger than the antibiotic alone [184]. Tsevelmaa et al. investigated the synergistic antibiotic potentiating activity of *Caryopteris mongolica* Bunge root extract against *B*. *melitensis* under in vitro conditions. The study showed that *C. mongolica* significantly reduced the neutrophil phagocytic activity of *B. melitensis* infected female BALB/c mice when used in combination with doxycycline compared to the antibiotic alone [185]. A study conducted by El-Tawab showed that plant extracts obtained from *Camellia sinensis*, *Thymus vulgaris*, *Zingiber officinale*, *Curcuma longa* and *Pelargonium graveolens* potentiated the antibacterial action of amoxicillin, gentamicin, doxycyciline, difloxacin against *Listeria monocytogenes*. *P. graveolens*-difloxacin combination indicated the highest anti-*Listeria* synergistic activity in the study, which induced an inhibition zone diameter of 8 mm larger than difloxacin alone [186].

**Table 2:** List of phytoextracts used in combination with antibiotics against *B. anthracis* and superbug bacteria for synergistic activity

Plant source	Compound	Antibiotic combination used with phytoextract	Effect/Mechanism of synergistic interaction on bacteria	References
*Stephania tetrandra*	Baicalin	β-lactam antibiotics Ciprofloxacin, Tetracycline	Reduction of β-lactamases in MRSA Inactivation of NorA, TetK efflux pumps expressed in *S. aureus*	[1, 207]
	Alkaloids, BisbenzylIsoquinoline, Demethyltetrandrine, Tetrandrine	Azithromycin, Ampicillin, Levofloxacin, Cefazolin	Reduces the MIC of MRSA	[282]
*Erythrina variegata*	Eryzerin-C/Erybraedin-A, Sophoraflavanone-G	Fosfomycin, Levofloxacin, Vancomycin, Gentamicin, Minocycline, Methicillin, Cefzonam	Inactivation of NorA efflux pumps expressed in MRSA and VRE	[226, 283]
Cranberry	Carvacrol	Erythromycin	Reduces MIC of Erythromycin-resistant Group A Streptococci	[284]
–		Tetracycline	Reduces the MIC of *S. aureus*	[207]
–	Conessine	Cefotaxime, Levofloxacin	Inactivation of MexAB‑OprM efflux pumps expressed in *P. aeruginosa*	[305]
*Artemisia annua*	Artemisinins	Penicillin G, Cefazolin, Ampicillin, Cefoperazone, Cefuroxime	Inactivation of AcrAB‑TolC efflux pumps expressed in *E.coli*	[229]
*Curcuma longa* Linn	Curcumin	Oxacillin, Norfloxacin	Reduces the MIC of MRSA	[264, 285]
		Cipofloxacin, Ampicillin		
		Gentamicin, Cefepime, Amikacin, Ampicillin, Ceftriaxone, Meropenem, Imipenem	Prevents or reduces biofilm formation in Gram-positive bacteria	[286]
		Ampicillin, Ciprofloxacin, Tetracycline, Vancomycin, Erythromycin, Gentamicin, Amikacin, Clindamycin, Fusidic acid, Penicillin	Reduces the MIC of MRSA and MSSA	[287]
		Plymyxin B, E	Inactivation of multi-drug resistant EtBr and EmrAB efflux pumps expressed in *A. baumannii*	[222, 223, 224]
*Hedychium coronarium*	Coronarin D	Tetracycline, Gentamicin, Rifampicin	Reduces the MIC of *B. cereus*	[191]
*Arctostaphylosuva-ursi*	Corilagin	Oxacillin, Cefmetazole	Reduces the fabrication of PBP2a in MRSA	[193]
*Jatropha elliptica*	2,6-Dimethyl-4-phenyl-pyridine-3,5-dicarboxylic acid diethyl ester	Erythromycin	Inactivation of NorA efflux pumps expressed in *S. aureus*	[227]
*Camellia sinensis* (Green tea)	Epigallocatechin gallate	Ampicillin or Sulbactam	Reduction of β-lactamases in MRSA	[202]
		Sulfamylon (mafenide acetate)	Reduces MIC of *A. baumannii* (Clinical isolate)	[164]
		Penicillin	Reduction of penicillinase in *S. aureus*	[288]
		Tetracycline	Inactivation of TetK efflux pumps expressed in *S. aureus*	[215, 217]
*Cytisus striatus*	Isoflavonoids	Erythromycin, Ciprofloxacin	Inactivation of NorA efflux pumps expressed in MRSA	[228]
–	Cathinone	Ciprofloxacin	Inactivation of acrAB‑TolC efflux pumps expressed in *S. typhimurium*	[230]
–	Theobromine	Ciprofloxacin, Tetracycline	Inactivation of acrAB‑TolC efflux pumps expressed in *S. typhimurium* and *K. pneumoniae*	
–	Myricetin	Cefoxitin	Inactivation of NorA efflux pumps expressed in MRSA	[289]
		Cefoxitin, Amoxicillin-clavulanate, Ampicillin-sulbactam	Inactivation of NorA efflux pumps in ESBL producing *K. pneumoniae* and MSSA	[290]
–	Rugosin B	Beta Lactam	Reduction of β-lactamases in MRSA	[1]
–	Totatrol	Methicillin	Reduces the synthesis of PBP2a in MRSA and MSSA	[1, 291]
Glycosmis	5-O-Methylglovanon	Ampicillin	Reduction of β-lactamases in *S. epidermidis* and *S. aureus*	[203]
*Punica granatum* (Pomegranate)	–	Gentamicin, Chloramphenicol, Tetracycline, Ampicillin, Oxacillin	Inactivation of NorA efflux pumps expressed in MRSA	[31]
*Herissantia tiubae*	Tiliroside	Lomefloxacin, Norfloxacin Cipofloxacin	Inactivation of NorA multi-drug resistant efflux pumps expressed in *S. aureus*	[221]
*Piper nigrum*	Piperine	Ofloxacin		[1]
Green tea	Epicatechin gallate	Oxacillin or Norfloxacin	Inactivation of multi-drug resistant efflux pumps expressed in *S. aureus* and MRSA	[1, 280, 293]
*Ipomoea murucoides*	Murucoidin	Norfloxacin	Reduces MIC of MRSA	[294]
*Rosmarinus officinalis*	Carnosic acid,	Erythromycin	Inactivation of NorA, MsrA efflux pumps expressed in *S. aureus*	[165]
–	Carnosol	Erythromycin, Tetracycline	Inactivation of NorA, MrsA, TetK efflux pumps expressed in *S. aureus*	[195]
–	Pinoresinol, Tiliroside, Momorcharaside B, Magnatriol B	β-Lactam antibiotics	Reduces the synthesis of PBP2a and PBP4 in MRSA MIC reduction and inactivation of CmeABC efflux pumps expressed in *C. jejuni*	[195]
–	p-Coumaric acid, Caffeic acid, Vanillic acid, Sinapic acid, Gallic acid, Taxifolin	Cipofloxacin, Erythromycin		[231]
*Persea lingue*	Kaempferol-3-O-α-L-(2,4-bis-E-pcoumaroyl) rhamnoside	Cipofloxacin	Reduces MIC of *S. aureus*	[214]
		Norfloxacin	Inactivation of NorA efflux pumps expressed in *S. aureus*	
–	Reserpine	Moxifloxacin, Sparfloxacin Ciprofloxacin	Inactivation of NorA efflux pumps expressed in *S. aureus*	[218]
–	Reserpine	Norfloxacin, Tetracycline, Ciprofloxacin	Inactivation of TetK, NorA, Bmr, MepA efflux pumps expressed in *S. aureus, S. pneumonia, B. subtilis*	[219, 220]
–	Tannic acid	Tetracycline, Norfloxacin	Inactivation of NorA, TetK efflux pumps expressed in *S. aureus*	[257]
*Berberis fremontii*	5'-methoxyhydnocarpin-D, Pheophorbide A	Amikicine, Ampicillin, Tetracycline	Inactivation of NorA multi-drug efflux pumps expressed in *S. aureus*	[36, 187, 292]
–	Theaflavin	Ampicillin	Reduces MIC of *S. maltophilia*	[181]
–	Tellimagrandin I	β-Lactam antibiotics	MIC reduction of MRSA	[1, 189, 194]
*Wrightia tinctoria*	Indoline, indirubin	Ciprofloxacin	Inactivation of NorA efflux pumps expressed in *S. aureus*	[212, 295]
*Alstonia scholaris*	–	Cephazolin	MIC reduction of *A. baumannii* ATCC 19,606	[165, 166]
*Adenium obesum*	–	Rifampicin		
*Cerbera odollam*	–	Cephazolin		
*Cerbera manghus*	–	Meropenem, Gentamicin,		
*Nerium oleander*	–	Erythromycin		
*Holarrhena antidysenterica*	–	Cephazolin		
*Plumeria obtusa*	–	Cephazolin, Rifampicin,		
*Wrightia pubescens*	–	Streptomycin		
*Thevetia peruviana*	–	Cephazolin, Rifampicin, Fusidic acid	MIC reduction of *A. baumannii* ATCC 19,606 and *P. chlororaphis*	[166]
*Jatropha curcas*	–	Ciprofloxacin, Cefotaxime, Rifampicin, Moxifloxacin, Ofloxacin	MIC reduction of *E. coli, P. monteilii, P. aeruginosa,* MRSA*, E. faecalis*, MDR *A. baumannii*	[180]
–	Catechol, Cinnamic acid, Ellagic acid, Ferulic acid, Gallic acid, Syringic acid, Tannic acid	Novobiocin	MIC reduction of *A. baumannii* JVC1053	[296]
	Ellagic acid, Tannic acid	Novobiocin, Coumermycin, Chlorobiocin, Rifampicin and Fusidic acid	Disruption of efflux pumps present in *A. baumannii* JVC1053	[225]
*Levisticum officinale*	–	Cipofloxacin	MIC reduction of *A. baumannii* NCTC 13,305	[171]
*Peganum harmala L,* *Cassis italic Mill,* *Carthamus tinctorius*	–	Ampicillin, Cefotaxime, Vancomycin, Chloramphenicol, Tetracyclines,	Increased inhibition zone diameter of antibiotic against *A. baumannii* (Clinical isolate)	[172, 173]
*Terminalia chebula*	–	Cefotaxime	Increased inhibition zone diameter of antibiotic against *A. baumannii* (Clinical isolate)	[167]
*–*	Berberine	Ciprofloxacin, Imipenem	MIC reduction of *A. baumannii* (Clinical isolate)	[88, 297]
		Vancomycin	MIC reduction and biofilm disruption of *C. difficile*	[306]
*Punica granatum,* *Holarrhena antidysenterica,* *Terminalia bellirica,* *Quisqualis indica,* *Terminalia* sp.*,* *Terminalia chebula*	–	Novobiocin	MIC reduction of *A. baumannii* ATCC 19,606	[168]
*Eucalyptus camaldulensis*	–	Ciprofloxacin Polymyxin B, Gentamicin,	MIC reduction of *A. baumannii* ATCC 19,606, *A. baumannii* ATCC BAA747, *A. baumannii* NCTC 13,420	[298]
*Allium sativum* (Garlic)	Allicin	Cefazolin, Oxacillin	MIC reduction of *S. aureus* and *S. epidermidis*	[158]
*–*	Rosmarinic acid –	Vancomycin, Amoxicillin, Ofloxacin	MIC reduction of *S. aureus* ATCC 25,923 and MRSA (Clinical isolate)	[177]
*Thymbra spicata* L	–	Ampicillin, Amikacin Cefotaxime, Ciprofloxacin	MIC reduction of *K. pneumoniae* and *S. aureus*	[307]
*Zingiber cassumunar*	–	Trimethoprim-sulfamethoxazole, Amoxicillin, Amoxicillin-clavulanic acid, Piperacillin-tazobactam, Ceftazidime, cefepime, Ceftriaxone, Imipenem, Cefotaxime, Meropenem, Ertapenem, Tetracygentamicin, Amikacin, Gentamicin, Amikacin, Doxycycline, Ciprofloxacin, Levofloxacin, Tetracycline	Increased inhibition zone diameter of antibiotic against XDR *A. baumannii*	[179]
*Holarrhena antidysenterica*	Conessine	Novobiocin, Rifampicin	Inactivation of AdeIJK multi-drug efflux pumps expressed in XDR *A. baumannii*	[207]
*Senna italica Mill*	–	Tetracyclines, Vancomycin, Ampicillin, Chloramphenicol	Increased inhibition zone diameter of antibiotic against *A. baumannii* (Clinical isolate)	[173]
*Capsicum* (chili peppers)	Capsaicin	Ciprofloxacin	Inactivation of NorA efflux pumps expressed in *S. aureus*	[213]
*Pithecellobium clypearia*	Gallic acid Methyl gallate Ethyl gallate Pyrogallol Myricetin-3-O-α-L-rhamnopyranoside Quercetin-3-O-α-L-rhamnopyranoside 5,3',4',5'-tetrahydroxy-flavan-7-gallate Epigallocatechin gallate	Imipenem, Cefoperazone, Ceftazidime, Levofloxacin, Amikacin, Tetracycline, Polymyxin B sulfate	MIC reduction of *A. baumannii*	[175]
	Oleanolic acid	Kanamycin, Gentamicin	MIC reduction *A. baumannii* ATCC 17,978	[178]
*Vaccinium macrocarpon* Aiton (Cranberry juice extract) *Ferula communis* *Plumbago zeylanica*	Proanthocyanidin	Levofloxacin		[196]
		Tetracycline	Reduces the synthesis of PBP2a in *H*. *pylori* Inactivation of AcrAB–TolC multi-drug efflux pumps expressed in *E. coli*	[308]
	Totarol and Ferulenol Plumbagin	Isoniazid	Inactivation of NorA efflux pumps expressed in *M. chelonei*, *M. smegmatis*, *M. intracellulare* and *M. xenopei*	[308]
–	Oleanolic acid	Rifampicin, Isoniazid, Ethambutol	MIC reduction of *M. tuberculosis* H37Rv (ATCC 27,294) and *M. tuberculosis* (Clinical isolate)	[309]
*Galenia africana* L	(E)-3,2′,4′-Trihydroxy-3′-methoxychalcone (2S)-5,7,2′-Trihydroxyflavonone	Isoniazid	MIC reduction of *M. tuberculosis* H37Rv	[310]
*Euclea natalensis* A.DC	7-Methyljuglone	Isoniazid, Rifampicin	MIC reduction of *M. tuberculosis* H37Rv	[311]
*Notopterygium incisum*	Isoimperatorin	Rifampicin	MIC reduction of *M. tuberculosis* H37Rv (ATCC 27,294) and *M. tuberculosis* (Clinical isolate)	[312]
*Piper nigrum* L	Piperine	Rifampicin	Inactivation of Rv1258c efflux pumps expressed in *M. tuberculosis* H37Rv (ATCC 27,294), Rifampicin-resistant *M. tuberculosis* and MDR *M. tuberculosis* (Clinical isolate)	[233]
*Knowltonia vesicatoria*	–	Isoniazid	MIC reduction of *M. tuberculosis* H37Rv	[313]
*Rehum palmatum,* *Glycyrrhiza glabra,* *Cassia angustifolia, Matricaria chamomilla,* *Chichorium intybus* *Alkanna tinctoria,* *Commiphora molmol,* *Curcuma aromatica,* *Ferula assa-foetida,* *Calligonum comosum,* *Rhamnus frangula* Cranberry juice		Ceftasidine, Gentamycin, Tobramycin, Spictinomycin, Cefoperazone	MIC reduction of *A. xylosoxidans* and *S. aureus*	[314]
		Nalidixic acid, Imipenem, Cefuroxime, Azithro-mycin, Colistin sulphate	Increased inhibition zone diameter of antibiotic against *E. coli, P. mirabilis, P. vulgaris, S. typhimurium* and *S. sonnei*	[315]
		Vancomycin, Tigecycline	Increased inhibition zone diameter of antibiotic against *E. faecalis, E. cloacae* and *S. aureus, E. faecium*	[316]
*Cocos nucifera*		Ampicillin, Amoxicillin, Chloramphenicol, Penicillin G, Tetracycline, Ciprofloxacin	MIC reduction of *E. coli, S. faecalis, L. ivanovii, V. vulnificus* and *V. fluvialis*	[317]
*Picralima nitida,* *Cassia arereh,* *Cylicodiscus gabunensis,* *Trichilia emetica*	-	Gentamicin, Erythromycin, Kanamycin	MIC reduction of *V. cholerae*	[183]
*Allium sativum* L. (Garlic)		Tetracycline, Penicillin, Rifampicin	Cell wall trauma and disruption of cell wall synthesis in *B. anthracis*	[49]
*Peganum harmala* L	Cinnamic acid, Ferulic acid, p-Coumaric acid	Novobiocin, Carbenicillin	Increased inhibition zone diameter of antibiotic against *B. anthracis* (Clinical isolate)	[182]
		Amikacin	Cell membrane trauma and disruption of cell membrane synthesis *S. aureus*	[196]
*Punica granatum* *Vangueria madagascariensis*	–	Ciprofloxacin	MIC reduction of *K. pneumoniae*	[242]
		Chloramphenicol, Ciprofloxacin	MIC reduction of *Acinetobacter* spp.	[243]
*Berberidaceae* spp.	Berberine +	Azithromycin	The MIC of berberine + azithromycin combination against MRSA was lowered by 50%-96.9% compared to the individual MICs of the two agents	[299]
	8‑acetonyl‑dihydroberbine	Levofloxacin	The ability to permeate the cell membrane of MRSA of 8‑acetonyl‑dihydroberbineis possibly superior than that of berberine	
*Stephania tetrandra*	Tetrandrine	Cefazolin	The MIC of tetrandrine + cefazolin combination against MRSA was lower by 75%–94% compared to the individual MICs of the two agents	[283]
	Demethyltetrandrine	Cefazolin	The MIC of demethyltetrandrine + cefazolin combination against MRSA was lower by 50%–94% compared to the individual MICs of the two agents	
*Thymus broussonetii*	Borneol/Carvacrol	Pristinamycin	The reduction of cellular PH level caused bacterial cell membrane disruption in *V. cholerae*, *K. pneumoniae*, *P. aeruginosa*	[300]
*Thymus maroccanus*	Thymol/carvacrol	Ciprofloxacin	MIC reduction of antibiotic against *V. cholerae*, *K. pneumoniae*, *P. aeruginosa*	[300]
*Zataria multiflora*	Thymol/carvacrol	Vancomycin	The MICs of thymol/carvacrol + vancomycin was lowered from 1 μg/mL to 0.125 μg/mL against *S. aureus* when used in combination	[301]
*Curcuma longa* Linn	Curcumin	Polymyxin B	The MIC of curcumin + polymyxin B combination against both clinical and non-clinical isolates of MRSA and *A. baumannii* was reduced by 3–tenfold compared to the individual MICs of the two agents	[222–224]
Horseradish root	Furanone C-30 + horseradish juice	Curcumin Tobramycin	Significantly reduced in vivo biofilm production in *P. aeruginosa* due to increased sensitivity to the antibiotic tested via anti-quorum sensing activity of horseradish juice and curcumin	[238]
*Oliveria decumbens*				
		Tetracycline, Oxacillin, Doxycycline	Increased inhibition zone diameter of antibiotic against *B*. *melitensis*	[184]
	Curcumin	Gentamicin Azithromycin	In vitro anti-biofilm activity mediated by anti-quorum sensing synergism with the selected antibiotics against *P. aeruginosa*. Plant-antibiotic combinations induced the reduction of autoinducer molecules and down regulation of virulent genes associated with quorum sensing receptors in *P. aeruginosa*	[239, 240]
	Baicalin + hamamelitannin, cinnamaldeyde	Vancomycin Clindamycin Tobramycin	Inhibition of biofilm formation in *B. cenocepacia*, *S. aureus* and *E. coli* in in vivo and in vitro assays and increased sensitivity to the antibiotics tested via quorum quenching ability of the isolated phytochemicals	[241]
-	Salicylate	Trimethoprim, Ciprofloxacin, chloramphenicol	Inactivation of ceoR efflux-pumps expressed in *B. cenocepacia*	[237, 318]

## Mechanism of synergistic activity of phytoextracts and antibiotic combined agents

**Fig. 3:**
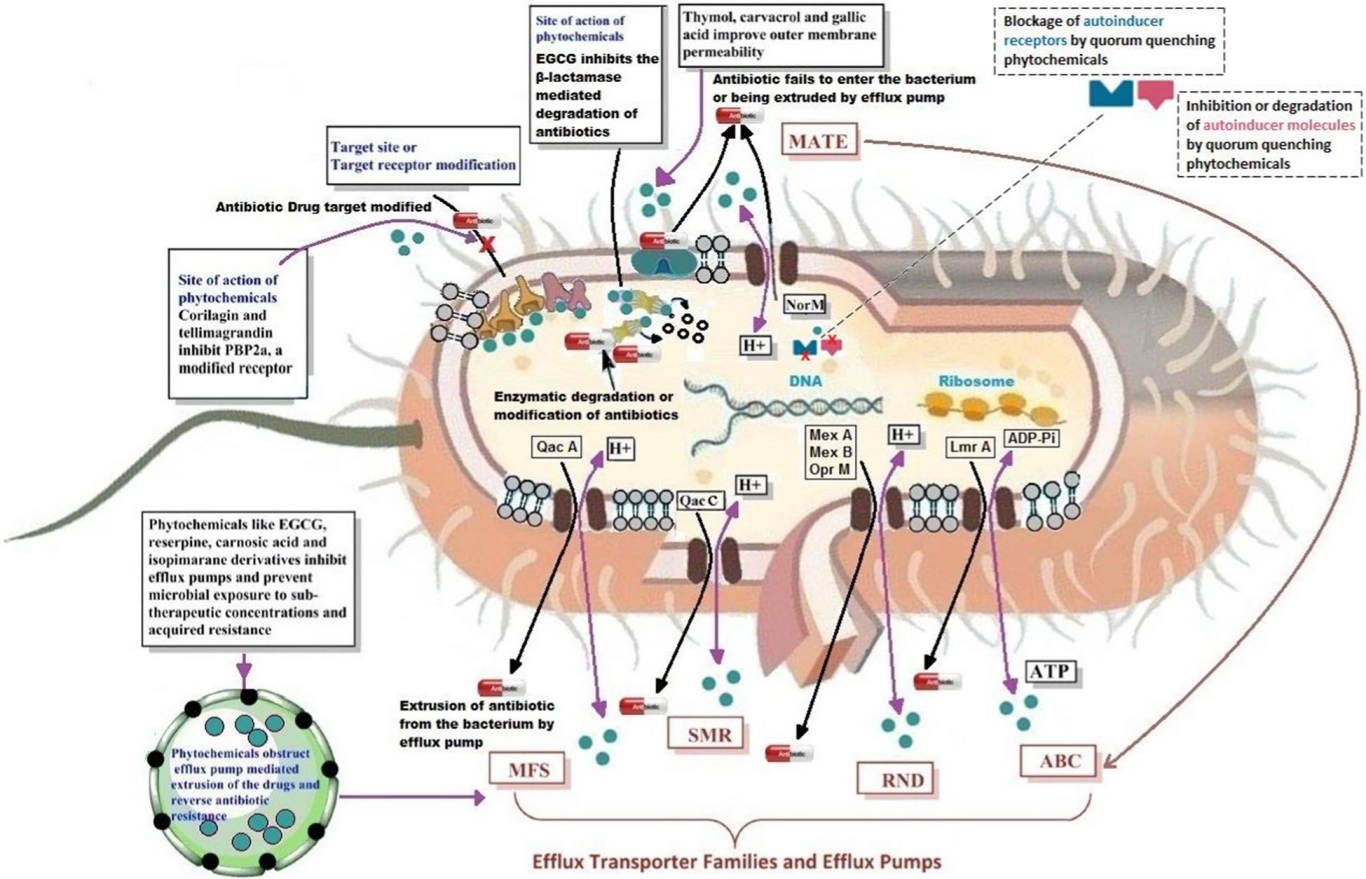
Mechanisms of action of phytochemical synergists in reversing antibiotic resistance in bacteria. Plant-derived compounds are capable of modifying the course of drug resistance in bacteria by interacting with antibiotic binding target sites/receptors, inhibition of antibiotic degrading enzymes, increasing cell membrane permeability to antibiotic molecules and disruption of drug extruding efflux pumps like MFS: Major Facilitator Super-family, SMR: Small Multidrug Resistance super-family, MATE: Multidrug and Aoxic Compound Extrusion super-family, RND: Resistance-nodulation-cell Division super-family, ABC: ATP-binding Cassette super-family. ATP: Adenosine triphosphate, ADP: Adenosine diphosphate, H + : Hydrogen, EGCG: Epigallocatechin gallate, PBP2a: Penicillin-binding protein 2a

Plant-derived compounds and agents combined with antibiotics have broad antibacterial activity against many types of bacteria. Several studies have indicated various antibacterial mechanisms of these combined compounds that highlight their ability to reverse antibiotic resistance. These mechanisms include the modification of active sites in the bacterial cell wall and the plasma membrane to increase the permeability of the antibiotic molecule, inhibition of extracellular enzymes that catalyze the modification or degradation of antibiotics, inactivation of efflux pumps to facilitate the intracellular accumulation of antibiotic molecules and disruption of quorum sensing signal molecules and their corresponding receptors (Fig. 3) [163]. Examples of phytoextract-antibiotic combinations and their synergistic effects/mechanisms against Gram-positive bacteria and *A. baumannii* are elucidated in Table 2.

### Plant-derived synergists as inhibitors of antibiotic binding site modification

Bacteria are capable of modifying the antibiotic binding target sites known as receptors (e.g. penicillin-binding proteins) to mediate antibiotic resistance. These alterations will no longer permit the binding of the antibiotic molecule to its specific receptor and permeate into the bacterial cell, rendering the antibiotic ineffective [187, 188]. Examples of this type of plant-derived synergistic compounds include corilagin, tellimagrandin I, pinoresinol, tiliroside, coronarin D, totatrol, baicalin, momorcharaside B and magnatriol B (Fig. 2) [1, 189–192]. Corilagin is a type of tannins isolated from *Arctostaphylos uva-ursi*, which indicates a MIC of 128 μg/mL against MRSA. However, the MIC dropped 2000 fold when used in combination with Oxacillin and β-Lactam antibiotics. Corilagin indicated strong synergism with an FICI of 0.5 with bactericidal action against MRSA [193]. Tellimagrandin I is another tannin compound that indicates a FICI of 0.39 for MRSA when used in combination with β-Lactam antibiotics. The combination of antibiotics and Tellimagrandin I had a MIC reduction of 128–512 fold when compared to the isolated phytochemical compound [194]. Phenolic compounds extracted from *Verbena officinalis*, *Magnolia officinalis*, *Daphne genkwa* and *Momordica charantia* such as pinoresinol, tiliroside, momorcharaside B, magnatriol B indicated a FICI of 0.375 for MRSA when used in combination with oxacillin. Bacteriological studies have indicated that these phytochemicals are capable of inhibiting PBP2a or PBP4 in MRSA [195]. Another study revealed that proanthocyanidin (Fig. 2) *isolated* from *Vaccinium macrocarpon* Aiton was able to synergistically interact with levofloxacin against *H. pylori*. Morphological investigations of the study revealed the reduction of PBP2a synthesis in *H. pylori* by proanthocyanidin [196]. A novel study revealed that garlic extracts obtained from the plant species *Allium sativum* L., which predominantly composed of phthalic acid (Fig. 2) and conceivably allicin showed synergistic antibiotic potentiating activity when used in combination with tetracycline, penicillin, rifampicin against the potential anthrax causing bio-agent *B. anthracis* strain Sterne 34F2. The indicative FICIs ranged from 0.5 to 0.8 for the selected plant-antibiotic combinations and microscopic analysis in the study detected garlic extract induced morphological disruptions on the cell wall of *B. anthracis* [49]. Isolated compounds like cinnamic acid, ferulic acid and p-coumaric acid are capable of inhibiting the synthesis of *S. aureus* cell membrane when combined with amikacin [197].

### Plant-derived synergists as inhibitors of antibiotic degrading/modifying enzymes

Certain bacteria can produce extracellular enzymes like β-lactamases and transacetylase that can chemically alter or even degrade antibiotic molecules. These enzymes can effectively retard the action of the antibiotic and render the antibiotic agent ineffective against the bacterium [33, 198–200]. However, studies have shown naturally occurring plant-based compounds that can synergistically interact with antibiotics to overcome these bacterial defenses. Examples of this type of phytochemical synergists include baicalin, rugosin B, 5-O-Methylglovanon and epigallocatechin gallate (Fig. 2) [1]. Baicalin extracted from *Scutellaria amoena* is one of the generally studied examples of plant-derived compounds contributing to this type of synergism, which was able to inhibit the activity of β-lactamases in MRSA and facilitated the antibacterial action of β-Lactam antibiotics [1, 201]. Epigallocatechin gallate is a polyphenolic compound that belongs to a class of catechin. An investigation revealed that epigallocatechin gallate (Fig. 2) isolated from tea extracts was able to reduce the MIC to 4 mg/L of ampicillin/sulbactam when used in combination with the antibiotic. The compound indicated a good FIC between 0.19 and 0.56 for MRSA and another study revealed that the compound had a FICI between 0.126 and 0.625 for 28 strains of MRSA. The study showed that epigallocatechin gallate can reduce the activity of penicillinase and β-lactamases in MRSA [202]. 5-O-methylglovanon isolated from *Glycosmis* plants is an isoprenyl flavonoid compound with broad-spectrum antibacterial activity. The compound can lower the production of β-lactamases to facilitate the action of ampicillin in *S. epidermidis* and *S. aureus* [203].

### Plant-derived synergists as inhibitors of active bacterial efflux pumps

Efflux pumps are among the most common bacterial defenses that lead to antibiotic resistance. These bacterial structures have the ability to extrude antibiotic molecules at a faster rate than the antibiotic can diffuse in the bacterial cell [204]. Efflux pumps are structurally present in Gram-positive and Gram-negative bacteria [205, 206]. There are several genes involved in the expression of these efflux pumps in Gram-positive and Gram-negative bacteria. Examples of such classes of genes include Tet, Acr, Ydh, Mex, Bla, Mdtef, and Nor [207]. These efflux pumps can be classified into five groups, depending on their capacity and drug extrusion mechanisms, such as MFS, SMR, MATE, RND and ABC (Fig. 3) [208]. Several studies have identified a number of plant-derived compounds that can counter the effects of these efflux pumps. Examples of such phytochemical synergists that modulated antibiotic resistance against Gram-positive bacteria include carnosic acid, carnosol, baicalin, erybraedin-a, sophoraflavanone-G, 2,6-dimethyl-4-phenyl-pyridine-3,5-dicarboxylic acid diethyl ester, myricetin, tiliroside, carnosic acid, carnosol, piperine, indoline, indirubin, capsaicin, kaempferol-3-o-α-l-(2,4-bis-E-pcoumaroyl) rhamnoside, reserpine, epicatechin gallate, 5'-methoxyhydnocarpin-D, pheophorbide A, isoflavonoids and tannic acid (Fig. 2) [1, 209–211]. Plant-derived synergists capable of modulating drug resistance facilitated by efflux pumps in *A. baumannii* include conessine and epigallocatechin gallate (Fig. 2).

The indoline compound indirubin isolated from *Wrightia tinctoria* indicated a high FICI of 0.45 for *S. aureus* SA199B when used in combination with ciprofloxacin. The compound was able to inhibit the NorA gene expressed efflux pump of the bacterium [212]. Capsaicin extracted from chili peppers (*Capsicum*) indicated similar synergistic action for *S. aureus* SA199 and *S. aureus* SA199B targeting their efflux pumps when used in combination with ciprofloxacin. Nevertheless, the compound reduced the MIC of ciprofloxacin by 2 to fourfold [213]. Carnosic acid and carnosol are terpenes isolated from *Rosmarinus officinalis* indicated a MIC of 64 μg/mL and 16 μg/mL respectively, against MDR *S. aureus*. However, the MIC decreased 32 fold for carnosol and 16 fold for carnosic acid when used in combination with erythromycin at a lower concentration of 10 μg/mL. It was found that their synergistic action was targeted at the *S. aureus* NorA efflux pumps [165]. Baicalin isolated from *Thymus vulgaris* L and *Scutellaria baicalensis* indicated synergistic action targeting NorA and TetK efflux pumps expressed in MRSA when used in combination with β-lactam antibiotics and tetracycline [201]. The flavonoid compound kaempferol rhamnoside demonstrated the ability to inhibit the activity of NorA efflux pumps in *S. aureus* when used in combination with ciprofloxacin and synergistically reduce the MIC by eightfold compared to the compound alone against the bacterium [214]. Epicatechin gallate (Fig. 2) is a type of catechin isolated from green tea extracts were able to interact synergistically with tetracycline to inactivate TetK and TetB efflux pumps expressing *Staphylococcus* spp. [215]. Another study indicated a fourfold reduction in MIC and efflux pump inhibitory activity in norfloxacin-resistant *S. aureus* when epicatechin gallate was used in combination with norfloxacin against the bacterium [216]. Epigallocatechin gallate isolated from *Camellia sinensis* potentiated the antibacterial activity of tetracycline against *S. aureus* by inhibiting the activity of TetK efflux pumps [215, 217]. The alkaloid compound reserpine was able to reduce the MIC of moxifloxacin, ciprofloxacin and sparfloxacin fourfold against *S. aureus*. Bacteriological studies indicated that reserpine was able to synergistically inhibit the activity of multi-drug efflux pumps expressed by the NorA gene in *S. aureus* [218]. Moreover, another study showed that reserpine was able to inactivate efflux pumps present in *S. aureus*, *S. pneumonia* and *B. subtilis* when used in combination with norfloxacin, tetracycline and ciprofloxacin [219, 220]. Recent investigations revealed that 5'-methoxyhydnocarpin-D and pheophorbide A (Fig. 2) isolated from *Berberis fremontii*, tiliroside isolated from *Herissantia tiubae* and piperine purified from *Piper nigrum* extracts were able to potentiate the antibacterial action of amikicine, ampicillin, tetracycline, lomefloxacin, norfloxacin and ofloxacin by inhibiting the activity of NorA multi-drug efflux pumps in *S. aureus* [1, 35, 221]. Phytoextracts obtained from *Punica granatum* (pomegranate) was able to potentiate the action of gentamicin, chloramphenicol, tetracycline, ampicillin, and oxacillin by inhibiting the activity of NorA efflux pumps in MRSA [31]. Another study indicated that conessine isolated from *Holarrhena antidysenterica* was able to synergistically potentiate the antibacterial action of novobiocin and rifampicin by inhibiting the activity of multidrug efflux pumps expressed by the AdeIJK gene in XDR *A. baumannii* [207]. A recent study revealed that curcumin (Fig. 2) purified and isolated from *Curcuma longa* Linn. was able to potentiate the antibacterial action of polymyxins when used in combination against MDR strains of *A. baumannii*. Curcumin-polymyxin B combinations indicated remarkably high FICIs of 0.156, 0.375, 0.068 for AB12, AB14, AB16 and NCTC 19606 strains of *A. baumannii* respectively. Bacteriological studies in the investigation elucidated that curcumin was able to reverse polymyxin resistance in MDR *A. baumannii* by modulating the activity of EtBr and EmrAB efflux pumps [222–224]. Furthermore, purified plant compounds like tannic acid and ellagic acid enhanced the antibacterial action of novobiocin, coumermycin, chlorobiocin, rifampicin and fusidic acid by reducing the MIC of each antibiotic by 2–fourfold. Bacteriological investigations in the study indicated that tannic acid and ellagic acid disrupted the activity of multidrug efflux pumps present in *A. baumannii* [225]. One study indicated that phytoextracts obtained from plants like *Erythrina variegata*, *Jatropha elliptica*, *Cytisus striatus* and *Persea lingue* also synergistically potentiated the action of antibiotics by inactivating efflux pumps present in drug-resistant Gram-positive bacteria like MRSA and VRE [1, 226–228]. Artemisinins (Fig. 2), an AcrAB‑TolC gene associated bacterial efflux pump disruptor isolated from *Artemisia annua* was able to potentiate the antibacterial action of penicillin G, cefazolin, ampicillin, cefoperazone and cefuroxime when used in combination against *E. coli,* which indicated FICIs of < 0.5 for each antibiotic [229]. Isolated compounds like Cathinone and Theobromine (Fig. 2) worked synergistically with ciprofloxacin and tetracycline when used in combination against *S. typhimurium* and *K. pneumoniae*. Both phytochemical compounds lowered the MICs of ciprofloxacin and tetracycline by 2–fourfold via the inhibition of efflux pumps expressed by AcrAB‑TolC gene [230]. Phenolic compounds like p-Coumaric acid, caffeic acid, vanillic acid, sinapic acid, gallic acid and taxifolin (Fig. 2) reduced the MICs of ciprofloxacin by 32 fold and erythromycin by 16 fold when used in combination against *C. jejuni*. These phenolic compounds disrupted the activity of efflux pumps in *C. jejuni* expressed by CmeABC gene [231]. Purified phytochemical compounds like ferulenol extracted from *Ferula communis* and plumbagin (Fig. 2) isolated from *Plumbago zeylanica* synergistically lowered the MIC of isoniazid against *Mycobacterium* spp. via the inhibition of efflux pumps expressed by NorA gene [232]. Furthermore, a study conducted by Sharma et al. revealed that piperine isolated from *Piper nigrum* L. (black pepper) reduced the MIC of rifampicin against the tuberculosis causing *M. tuberculosis* H37Rv strain by 4–eightfold. Bacteriological studies indicated that piperine was able to synergistically inhibit the activity of multi-drug efflux pumps expressed by the Rv1258c gene in *M. tuberculosis* [233]. Catechin (Fig. 2) compounds, epigallocatechin and epicatechin gallates were the first herbal drugs to receive FDA approval in 2006. The leaf extract of *Camellia sinensis* consists of about 85% to 95% catechins and the essence of the plant was used in the topical management and treatment of genital warts. [234]. Curcumin isolated from *Curcuma longa* Linn. is another FDA approved plant based natural product that has proven benefits in clinical trials, and capable of potentiating synthetic antibiotics when used in combination with polymyxin B against MRSA and *A. baumannii* [222–224]. Cranberry juice extract of *Vaccinium macrocarpon* Aiton which is abundant of proanthocyanidin also received FDA approval for treating uropathogenic *E. coli* [235, 236]. A derivative phytochemical compound known as salicylate (Fig. 2) mediated synergistic action against the pulmonary pathogen *Burkholderia cenocepacia* when used in combination with trimethoprim, ciprofloxacin and chloramphenicol. The study indicated that salicylate reduced the MIC of the selected antibiotics by tenfold and the combinations potentially inhibited the activity of efflux-pumps in *B. cenocepacia* [237].

### Plant-derived synergists as biofilm formation/quorum sensing antagonists

Plant-derived synergists are also capable of negating the process of quorum sensing in bacteria. A novel study conducted by Christensen et al. indicated that horseradish juice and curcumin supplemented with furanone C-30 was able to induce significant synergistic quorum quenching activity when combined with tobramycin against *P. aeruginosa* PAO1 in female BALB/c mice [238]. Bacteriological studies indicated that curcumin and phytochemicals from horseradish juice reduced of the secretion of autoinducer molecules like C-4 and C12- homoserine lactones from the bacterium. A similar study detected In vitro synergistic anti-quorum sensing activity of curcumin with gentamicin and azithromycin combinations against *P. aeruginosa*. The study also concluded that plant-antibiotic combinations were able to reduce the activity of N-acyl-homoserine lactone autoinducer signaling molecules and down regulate virulent genes like rhlA, lasB and rhl associated with quorum sensing in *P. aeruginosa* [239, 240]. Furthermore, synergistic anti-biofilm and anti-quorum sensing activities were detected when phytochemical compounds baicalin, hamamelitannin and cinnamaldehyde (Fig. 2) were combined with vancomycin, clindamycin and tobramycin against clinical isolates of *B. cenocepacia, S. aureus* and *E. coli* in both in vitro assays and in greater wax moth (*Galleria mellonella*), *Caenorhabditis elegans* nematode and female BALB/c mice models used in in vivo assessments [241]. Moreover, naturally synthesized phytochemical compounds like furanones, particularly the halogenated variant known as (5Z)-4-bromo-5-(bromomethylene)-3-butyl-2(5H)-furanone (Fig. 2) was able to attenuate the QS system of *B. anthracis* [319, 320].

## Concluding remarks and future perspectives

The problem of antibiotic resistance is growing rapidly, and the prospects for the application of antibiotic agents in the future have reached uncertainty. Despite the mass production of antibiotics by the pharmaceutical industries in recent decades, bacteria have shown greater resistance to these antibiotics. Plants are remarkable and phenomenal sources of new bioactive compounds with broad-spectrum antibacterial properties. These compounds can assign direct action or interact synergistically with antibiotics to work against bacteria. The following review summarizes the findings of recent investigations based on phytoextracts in combination with existing antibiotics in the context of their drug resistance modulating potential against the anthrax causative organism *Bacillus anthracis* and MDR and XDR strains of emerging bacterial superbugs. Phytochemical-antibiotic combinations have shown promising results as agents with different mechanisms for modifying and reversing antibiotic resistance. For instance, phytochemicals such as epigallocatechin gallate can interact synergistically with different classes of antibiotics. Depending on the bacterium, this compound can mediate synergism and increase the potency of antibiotics, deactivating β-lactamases and multidrug efflux pumps. Pre-clinical studies have shown that these synergistic compounds can significantly reduce the MIC of bacteria when used in combination with antibiotics. The motivation in antimicrobial synergy research leads to the discovery and production of new antimicrobial agents. However, the underlying action mechanisms of synergistic compounds have not yet been fully explored. A profile that indicates a complete understanding of the pharmacokinetics and pharmacodynamics of the combination agents are required to qualify as a standardized and effective antimicrobial drug. Furthermore, in vivo and nano-medicine drug delivery studies based on combined synergists of plant compound-antibiotics can be deployed for better understand the toxicological responses and bioavailability of the combined agents, to determine their true relevance and safety in the treatment of bacterial infections in humans. Advanced techniques such as isobolograms and phytochemical paradigms can be used to analyze and utilize regions of synergistic interaction between mixtures of antibacterial drugs. At present, the availability of experimental data based on antibiotic-potentiating mechanisms of plant synergists against *Bacillus anthracis* and antibiotic resistance modulating effects of plant based QS antagonists are limited and therefore, broadening of these studies are imperative. Furthermore, it is necessary to exploit drug resistance modulating potentials of novel combinative products focusing on plant-derived antibodies and antibiotics against bacterial superbugs and *B. anthracis*. The efficiency of plant-antibiotic synergists and their drug resistance modulating mechanisms are needed to be investigated on recently CDC listed superbugs like *Bordetella pertussis* and *Mycoplasma genitalium* and other infectious disease causing pathogens like *Rickettsia rickettsii*, *Neisseria* spp., *Yersinia pestis* and *Francisella tularensis*.

## Data Availability

The following review was based on data extracted from published research article available in all relevant databases with no limitation up to 10th January 2021.

## Abbreviations

β: Beta
MIC: Minimum inhibitory concentration
MRSA: Methicillin-resistant *Staphylococcus aureus*
MSSA: Methicillin-sensitive *Staphylococcus aureus*
VRE: Vancomycin-resistant *Enterococcus faecium*
PBP: Penicilin-binding protein
*S. aureus*: *Staphylococcus aureus*
*S. pneumonia*: *Streptococcus pneumoniae*
*B. subtilis*: *Bacillus subtilis*
*S. epidermidis*: *Staphylococcus epidermidis*
FICI: Fractional Inhibitory Concentration Index
μg/mL: Microgram per milliliters
μg/μL: Microgram per microliters
*L. monocytogenes*: *Listeria monocytogenes*
*E. faecium*: *Enterococcus faecium*
*O. oeni*: *Oenococcusoeni*
*L.*hilgardii: *Lactobacillus hilgardii*
*S. agalactiae*: *Streptococcus agalactiae*
*S. pyogenes*: *Streptococcus pyogenes*
*B. cereus*: *Bacillus cereus*
*B. anthracis*: *Bacillus anthracis*
*A. baumannii*: *Acinetobacter baumannii*
*P. aeruginosa*: *Pseudomonas aeruginosa*
*E. coli*: *Escherichia coli*
*S. typhimurium*: *Salmonella typhimurium*
*K. pneumoniae*: *Klebsiella pneumoniae*
*C. jejuni*: *Campylobacter jejuni*
*S. pneumonia*: *Streptococcus pneumoniae*
*S. maltophilia*: *Stenotrophomonas maltophilia*
*P. monteilii*: *Pseudomonas monteilii*
*P. chlororaphis*: *Pseudomonas chlororaphis*
*C. difficile*: *Clostridium difficile*
*H. pylori*: *Helicobacter pylori*
*M. chelonei*: *Mycobacterium chelonae*
*M. smegmatis*: *Mycobacterium smegmatis*
*M. intracellulare*: *Mycobacterium intracellulare*
*M. xenopei*: *Mycobacterium xenopei*
*M. tuberculosis*: *Mycobacterium tuberculosis*
*A. xylosoxidans*: *Achromobacter xylosoxidans*
*P. mirabilis*: *Proteus mirabilis*
*P. vulgaris*: *Proteus vulgaris*
*S. sonnei*: *Shigella sonnei*
*E. cloacae*: *Enterobacter cloacae*
*S. faecalis*: *Streptococcus faecalis*
*L. ivanovii*: *Listeria ivanovii*
*V. vulnificus*: *Vibrio vulnificus*
*V. fluvialis*: *Vibrio fluvialis*
*V. cholerae*: *Vibrio cholerae*
*B. cenocepacia*: *Burkholderia cenocepacia*
*B. melitensis*: *Brucella melitensis*
MFS: Major facilitator super-family
SMR: Small multidrug resistance super-family
MATE: Multidrug and aoxic compound extrusion super-family
RND: Resistance-nodulation-cell division super-family
ABC: ATP-binding cassette super-family
mg/mL: Milligram per milliliter
DNA: Deoxyribonucleic acid
RNA: Ribonucleic acid
ATP: Adenosine triphosphate
sp.: Species (single)
spp.: Species (multiple)
ESBL: Extended-spectrum beta-lactamases
ESKAPE: *Enterococcus faecium*, *Staphylococcus aureus*, *Klebsiella pneumoniae*, *Acinetobacter baumannii*, *Pseudomonas aeruginosa*, And *Enterobacter* species
NADH: Nicotinamide adenine dinucleotide
MIC: Minimum inhibitory concentration
ADP: Adenosine diphosphate
H +: Hydrogen
EGCG: Epigallocatechin gallate
PBP2a: Penicillin-binding protein 2a
WHO: World Health Organization
CDC: Centers for Disease Control and Prevention
FDA: Food and Drug Administration
ATCC: American Type Culture Collection
NCTC: National Collection of Type Cultures
SPI: Salmonella pathogenicity island
MDR: Multidrug-resistant
XDR: Extensively drug-resistant
PDR: Pandrug-resistant
QS: Quorum sensing
